# ADNEX risk prediction model for diagnosis of ovarian cancer: systematic review and meta-analysis of external validation studies

**DOI:** 10.1136/bmjmed-2023-000817

**Published:** 2024-02-17

**Authors:** Lasai Barreñada, Ashleigh Ledger, Paula Dhiman, Gary Collins, Laure Wynants, Jan Y Verbakel, Dirk Timmerman, Lil Valentin, Ben Van Calster

**Affiliations:** 1 Department of Development and Regeneration, KU Leuven, Leuven, Belgium; 2 Nuffield Department of Orthopaedics, Rheumatology and Musculoskeletal Sciences, University of Oxford Centre for Statistics in Medicine, Oxford, UK; 3 Department of Epidemiology, Universiteit Maastricht Care and Public Health Research Institute, Maastricht, Netherlands; 4 Department of Public Health and Primary care, KU Leuven, Leuven, Belgium; 5 Nuffield Department of Primary Care Health Sciences, University of Oxford, Oxford, UK; 6 Leuven Unit for Health Technology Assessment Research (LUHTAR), KU Leuven, Leuven, Belgium; 7 Department of Obstetrics and Gynaecology, UZ Leuven campus Gasthuisberg Dienst gynaecologie en verloskunde, Leuven, Belgium; 8 Department of Obstetrics and Gynaecology, Skåne University Hospital, Malmo, Sweden; 9 Department of Clinical Sciences Malmö, Lund University, Lund, Sweden; 10 Department of Biomedical Data Sciences, Leiden University Medical Centre, Leiden, Netherlands

**Keywords:** Obstetrics, Statistics

## Abstract

**Objectives:**

To conduct a systematic review of studies externally validating the ADNEX (Assessment of Different Neoplasias in the adnexa) model for diagnosis of ovarian cancer and to present a meta-analysis of its performance.

**Design:**

Systematic review and meta-analysis of external validation studies

**Data sources:**

Medline, Embase, Web of Science, Scopus, and Europe PMC, from 15 October 2014 to 15 May 2023.

**Eligibility criteria for selecting studies:**

All external validation studies of the performance of ADNEX, with any study design and any study population of patients with an adnexal mass. Two independent reviewers extracted the data. Disagreements were resolved by discussion. Reporting quality of the studies was scored with the TRIPOD (Transparent Reporting of a multivariable prediction model for Individual Prognosis Or Diagnosis) reporting guideline, and methodological conduct and risk of bias with PROBAST (Prediction model Risk Of Bias Assessment Tool). Random effects meta-analysis of the area under the receiver operating characteristic curve (AUC), sensitivity and specificity at the 10% risk of malignancy threshold, and net benefit and relative utility at the 10% risk of malignancy threshold were performed.

**Results:**

47 studies (17 007 tumours) were included, with a median study sample size of 261 (range 24-4905). On average, 61% of TRIPOD items were reported. Handling of missing data, justification of sample size, and model calibration were rarely described. 91% of validations were at high risk of bias, mainly because of the unexplained exclusion of incomplete cases, small sample size, or no assessment of calibration. The summary AUC to distinguish benign from malignant tumours in patients who underwent surgery was 0.93 (95% confidence interval 0.92 to 0.94, 95% prediction interval 0.85 to 0.98) for ADNEX with the serum biomarker, cancer antigen 125 (CA125), as a predictor (9202 tumours, 43 centres, 18 countries, and 21 studies) and 0.93 (95% confidence interval 0.91 to 0.94, 95% prediction interval 0.85 to 0.98) for ADNEX without CA125 (6309 tumours, 31 centres, 13 countries, and 12 studies). The estimated probability that the model has use clinically in a new centre was 95% (with CA125) and 91% (without CA125). When restricting analysis to studies with a low risk of bias, summary AUC values were 0.93 (with CA125) and 0.91 (without CA125), and estimated probabilities that the model has use clinically were 89% (with CA125) and 87% (without CA125).

**Conclusions:**

The results of the meta-analysis indicated that ADNEX performed well in distinguishing between benign and malignant tumours in populations from different countries and settings, regardless of whether the serum biomarker, CA125, was used as a predictor. A key limitation was that calibration was rarely assessed.

**Systematic review registration:**

PROSPERO CRD42022373182.

**WHAT IS ALREADY KNOWN ON THIS TOPIC**

- The optimal management of ovarian tumours depends on tumour type, and therefore diagnosis before surgery is important to determine an appropriate treatment plan
- ADNEX (Assessment of Different Neoplasias in the adnexa) is a diagnostic prediction model that estimates the probability that a tumour is benign, borderline, stage I primary invasive, stage II-IV primary invasive, or secondary metastatic
- ADNEX has two versions: one with and one without the serum biomarker, cancer antigen 125 (CA125)

**WHAT THIS STUDY ADDS**

- Both versions of ADNEX differentiated between benign and malignant tumours, with an area under the receiver operating characteristic curve of ≥0.90 for different populations, countries, and settings
- ADNEX with CA125 was superior to ADNEX without CA125 for distinguishing between malignant subtypes, with moderate heterogeneity for the models' performance
- The models had a 95% (with CA125) and 91% (without CA125) chance of being of use clinically in new centres when used at the 10% risk of malignancy threshold
- Many studies included in this review and meta-analysis had poor reporting and unjustified exclusion of patients with missing data, and calibration performance was not assessed

**HOW THIS STUDY MIGHT AFFECT RESEARCH, PRACTICE, OR POLICY**

- ADNEX can be used to support clinical decisions
- ADNEX without CA125 is sufficient to help decide whether conservative follow-up surgery in a local centre or referral to an oncology centre is appropriate
- To help decide on optimal management of a tumour suspected to be malignant, ADNEX with CA125 is superior to ADNEX without CA125, because it differentiates better between malignant subtypes
- The methodological quality of external validation studies needs to improve in terms of justification of sample size, handling of missing data, and assessing calibration of the model

## Introduction

The optimal management of patients with an ovarian mass depends on the histology of the mass. Patients with a benign mass can be managed without surgery, with clinical and ultrasound follow-up, or with conservative surgical techniques.^1 2^ Malignant tumours benefit from management in specialised oncology centres, but borderline malignancies, stage I primary invasive tumours, and advanced primary invasive tumours might require different surgical approaches.^3 4^ To optimise patient triage without operating on all masses, diagnostic models can be used to estimate the likelihood of malignancy and hence to plan treatment for patients.

Given the potential advantages of accurately predicting the risk of malignancy, the International Ovarian Tumour Analysis (IOTA) group developed the Assessment of Different Neoplasias in the adnexa (ADNEX) risk prediction model, based on three clinical and six ultrasound predictor variables.^5^ The clinical variables are age, serum levels of the biomarker, cancer antigen 125 (CA125), and type of centre (oncology centre *v* other). An oncology centre is defined as a tertiary referral centre with a specific gynaecology oncology unit. The ultrasound variables are the maximum diameter of the lesion, proportion of solid tissue (defined as the largest diameter of the largest solid component divided by the largest diameter of the lesion), number of papillary projections, presence of >10 cyst locules, presence of acoustic shadows, and ascites. The ADNEX multinomial logistic regression model estimates the risk of five tumour types: benign, borderline, stage I primary invasive, stage II-IV primary invasive, and secondary metastatic.

The total risk of malignancy calculated by ADNEX is the sum of the risks for each malignant subtype. ADNEX has two versions: one with and one without CA125 as a predictor (the ADNEX formulas are provided in online supplemental material S1).^5^ When we refer to the ADNEX model or ADNEX, we refer to both versions of the model. The model was developed on data from 5909 patients with an adnexal mass who subsequently underwent surgery, recruited at 24 centres in 10 countries (Belgium, Italy, Czech Republic, Poland, Sweden, China, France, Spain, UK, and Canada). Although developed on data from patients that underwent surgery, the performance of ADNEX has also been evaluated in cohorts that included patients managed without surgery.^6–9^

10.1136/bmjmed-2023-000817.supp1 Supplementary data

ADNEX is included in national guidelines (eg, in Belgium, the Netherlands, and Sweden),^10–12^ and recommended by scientific societies, such as the International Society of Ultrasound in Obstetrics and Gynecology, European Society of Gynaecological Oncology, European Society for Gynaecological Endoscopy, and the American College of Radiology.^4 13^ Also, manufacturers of ultrasound machines have begun to incorporate ADNEX directly into their machines.

Several external validation studies of ADNEX have been carried out. So far, five published systematic reviews and meta-analyses of ADNEX have summarised 3-22 external validation studies.^14–18^ All of the systematic reviews evaluated ADNEX only as a diagnostic test, reporting a summary sensitivity and specificity at a threshold for the estimated risk of malignancy of 10% or 15%.^14–18^ The ADNEX model not only classifies masses as benign or malignant, however, it can also be used as a risk prediction model, providing probability estimates for five different tumour types at the individual patient level. Hence focusing only on its performance in classifying tumours, risks losing useful information.^19^ When validating ADNEX as a diagnostic test at a 10% threshold, the performance metrics (ie, sensitivity and specificity) do not take into account the individual risks predicted but the same weight is given to a misclassified patient with an 11% risk as with a 99% risk. This approach means that these meta-analyses have not fully validated the diagnostic performance of ADNEX; for example, pooling discrimination performance (area under the receiver operating characteristic curve, AUC) allows determination of the ability of the model to differentiate between patients with and without the outcome across different thresholds. Guidelines on how to evaluate the quality and risk of bias of external validation studies of risk prediction models have been produced.^20–22^ These guidelines should be used in meta-analyses of validation studies. Hence the objectives of this study were to perform a systematic review of studies that externally validated ADNEX, to describe reporting completeness and risk of bias of the validation studies, and to conduct meta-analyses of measures of performance of the model.

## Methods

### Protocol registration

We report this study according to the PRISMA (Preferred Reporting Items for Systematic Reviews and Meta-Analyses, online supplemental file 2) and TRIPOD-SRMA (Transparent Reporting of multivariable prediction models for Individual Prognosis Or Diagnosis: checklist for Systematic Reviews and Meta-Analyses, online supplemental file 3) checklists.^22 23^

10.1136/bmjmed-2023-000817.supp2 Supplementary data

10.1136/bmjmed-2023-000817.supp3 Supplementary data

### Eligibility criteria

Any study that carried out an external validation to evaluate the performance of the ADNEX model, based on any study design and any study population, was eligible for inclusion in the systematic review. Exclusion criteria were studies that did not evaluate the performance of the ADNEX model; studies that only evaluated the predictive performance of updated versions of ADNEX; studies where only an abstract was available, or the full text could not be obtained; and case studies presenting the performance of ADNEX for individual patients (this criterion was not prespecified in the protocol but was added post hoc on review of the search results). Updating can refer to recalibration, refitting, or extension with additional predictors.^24^ Studies that conducted comparisons of ADNEX with other models, reporting performance metrics, were eligible for inclusion.

### Information sources and search strategy

We created a search string and overall search strategy with the help of biomedical reference librarians from the KU Leuven Libraries. We searched the electronic databases Medline (through PubMed), Embase, Web of Science, and Scopus for published articles, and Europe PMC for preprints. The search dates were from the publication of the first ADNEX paper (15 October 2014) to 15 May 2023 (date when the final search was run). We also screened all articles citing the original ADNEX paper.^5^ The reference lists of relevant review and opinion articles retrieved by the search strings were checked for other potentially eligible articles. Forward and backward snowballing (forward and back cross reference checking) of the included articles was performed to identify additional publications.^25^ Language was not restricted, but for papers in languages other than English, Spanish, Dutch, French, or Swedish, we used an automatic translation tool (deepl.com) to decide whether to include a paper and to extract information. Online supplemental material S2 shows the full search strategy.

### Study selection

The studies we identified in our search were imported into Zotero reference manager, where they were automatically deduplicated. The deduplicated records were then imported into the Rayyan web application for manual deduplication (by LB) and subsequent screening of the title and abstract by two independent authors. Disagreements were resolved by discussion between the two authors (LB and AL).^26^

Three of the authors (BVC, LV, and DT) were members of the IOTA group that developed ADNEX, so we divided the studies into those that were linked or not linked to IOTA. A study was linked to IOTA if it was coauthored by a member of the IOTA steering committee (online supplemental material S3). IOTA linked papers, as well as a few others with a potential conflict of interest (ie, including authors that are or were IOTA collaborators), were independently assessed by two of the authors (PD and GSC, medical statisticians with expertise in prediction modelling and unrelated to IOTA). All other studies were independently assessed (by LB and AL). Disagreements were resolved by discussion between reviewers, and for the non-IOTA papers, by discussion with authors BVC, LV, and JYV.

### Data extraction and data items

Data were extracted and entered into a standardised data extraction form in Microsoft Excel. Data extraction focused on the general and design characteristics of the studies, target population, reference standard, sample size, performance results, reporting quality, methodological quality, and risk of bias (online supplemental table S1). The extraction form was adapted from the CHARMS (CHecklist for critical Appraisal and data extraction for systematic Reviews of prediction Modelling Studies) and TRIPOD (Transparent Reporting of a multivariable prediction model for Individual Prognosis Or Diagnosis) tools, and PROBAST (Prediction model Risk Of Bias Assessment Tool).^21 27 28^

To describe the performance of the model, we extracted information on any reported measure related to discrimination, calibration, diagnostic accuracy, or clinical utility. The reference standard could be binary (eg, benign *v* malignant) or multinomial (eg, the five tumour types predicted by ADNEX). Performance data were extracted for all reported validations (ie, for ADNEX with and without CA125), subgroup analyses, sensitivity analyses, and for multicentre studies, results specific to each centre. For each study, we assessed the reporting of all TRIPOD items that were applicable to the external validation studies (online supplemental table S2). We also checked PROBAST’s signalling questions and evaluated risk of bias for each subdomain (participants, predictors, outcome, and analysis) and overall. We included our rationale for classification of the risk of bias.

We contacted study authors to obtain further information or results when centre specific results were not reported in multicentre studies, type of centre was not explicitly reported (if no response, the clinical coauthors, JYV, DT, and LV, classified the centre), overall performance was reported but not performance by menopausal status, or performance was not reported for patients who underwent surgery separately in studies that included patients who were managed surgically and non-surgically. Online supplemental table S1 and Open Science Framework repository (extraction sheet, https://osf.io/jtsvd/) have details on all extracted items.^29^

### Statistical analysis and quantitative data synthesis

Data were summarised with descriptive statistics and data visualisations. Meta-analysis of performance, based on specific results for each centre in multicentre studies, where possible, was conducted with the random effects meta-analysis method. Meta-analysis was done separately for ADNEX with and without CA125. We used 95% confidence intervals for the summary performance and assessed heterogeneity with τ^2^ and 95% prediction intervals. Online supplemental material S4-6 have details on the statistical methodology, including meta-analysis methods, explanations of net benefit and relative utility, and assessment of publication bias.

Meta-analysis of the AUC for benign versus malignant tumours was done on the logit scale. Meta-analysis for sensitivity and specificity was also performed on the logit scale with a random effects meta-analysis.^30^ Because the meta-analysis was conducted only for the 10% threshold for the risk of malignancy, we did not need the 95% confidence ellipse in receiver operating characteristic curve space, so we did not use the bivariate random effects model as specified in the protocol. The 10% threshold was most commonly used in the articles included in our systematic review, and is a commonly recommended threshold.^4^ Meta-analysis of net benefit^31^ and relative utility^32^ at the 10% risk of malignancy threshold was performed with bayesian trivariate random effects meta-analyses of sensitivity, specificity, and prevalence of malignancy.^33^ For bayesian methods, 95% credible intervals are reported instead of 95% confidence intervals. With the bayesian approach, the probability that the model is useful in a new centre can be estimated (ie, the probability that relative utility is >0). To deal with multinomial discrimination performance, we conducted a meta-analysis of AUC values between pairs of tumour outcomes (pairwise AUC values) on a logit scale. We only included studies that used the conditional risk method to calculate pairwise AUC values.^34^

Subgroups were defined based on geographical location, type of centre, and menopausal status. Sensitivity analyses were based on judgment of the risk of bias and on whether the study was linked to IOTA. As prespecified in the protocol, we only conducted a meta-analysis of performance if at least three estimates in a specific analysis could be retrieved from the included studies. To assess the association between prevalence of malignancy and AUC, and sensitivity and specificity at the 10% risk of malignancy threshold, we used meta-regression.^35^

Reporting bias and small study effects were visually explored with funnel plots adapted for the AUC. The body of evidence was assessed with an adapted version of GRADE (Grading of Recommendations Assessment, Development and Evaluation).^36^ All analyses were performed in R version 4.2.2 with the package metamisc for AUC, meta and mada for sensitivity and specificity, and rjags for net benefit and relative utility.^37–40^ Bayesian methods were computed with JAGS version 4.3.1.^40^

### Patient and public involvement

Patients and the public were not involved in the design, or conduct, or reporting, or dissemination plans of this research. Preliminary results of the research were presented at the ISUOG World Congress in Seoul (October 2023). As this study was a systematic review, there was no collection of patients' data, and we use only the information publicly available in the published papers.

## Results

**Figure 1:**
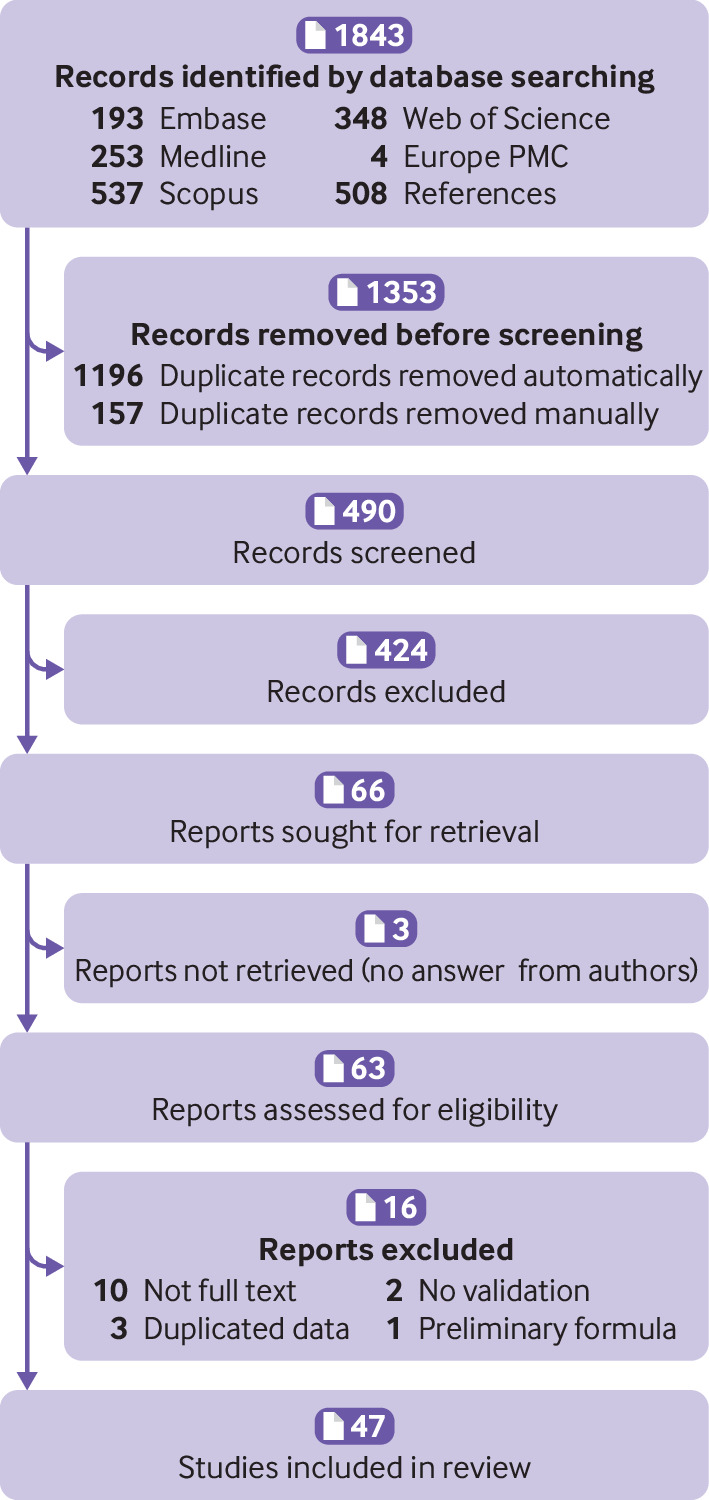
PRISMA (Preferred Reporting Items for Systematic Reviews and Meta-Analyses) flowchart of study inclusions and exclusions

We identified 1843 records and screened 490 after duplicates were removed. Forty seven studies met our inclusion criteria and were included in this systematic review (figure 1 and online supplemental table S3).^6–9 41–83^ Three studies were excluded because the same data were used in another included study, and one study was excluded because a preliminary formula of ADNEX was used.^84–87^ The data of three studies that were linked to IOTA and three other studies with a potential conflict of interest were extracted by authors PD and GSC.^6 50 63 72 74 75^

Table 1 summarises the key study characteristics and online supplemental table S3 shows the specific details for each study. The unit of analysis was the patient in 42 (89%) studies and the tumour in five (11%) studies. When tumour was the unit of analysis, multiple tumours for the same patient could be included. The 47 studies reported on 17 007 tumours, with a median study sample size of 261 tumours (range 24-4905). Validations were conducted in 28 countries, with most studies conducted in Asia (51%) and Europe (38%). Online supplemental material S7 gives a list of reporting inconsistencies. All studies classified borderline tumours as malignant tumours.

**Table 1:** Characteristics of 47 included studies

Study characteristics	No (%)	Comments
Unit of analysis		
Patient	42 (89)	1 tumour per patient
Tumour	5 (11)	>1 tumour per patient possible
No of countries in region		
Asia	24 (51)	Common countries (No of studies): China (12), South Korea (3)
Europe	18 (38)	Common countries (No of studies): Poland (7), Italy (4), Spain (4), UK (3), and Sweden (3)
South America	3 (6)	—
North America	2 (4)	—
No of centres		
1	37 (79)	—
2-5	7 (15)	—
>5	3 (6)	Range 8-17
Type of centre		
Oncology centres	35 (74)	—
Non-oncology centres	2 (4)	—
Both types of centres	4 (9)	—
Unclear	6 (13)	—
ADNEX version (No of studies)		
ADNEX with CA125	37 (79)	21 only used ADNEX with CA125, 16 used both
ADNEX without CA125	19 (40)	3 only used ADNEX without CA125, 16 used both
Mixed	3 (6)	ADNEX with CA125 used if CA125 was available
Unclear	4 (9)	—
Selection based on histology (No of studies)		
No	39 (83)	—
Yes	8 (17)	For example, borderline tumours excluded, only invasive tumours
Focus only on clinical subgroup (No of studies)		
No	42 (89)	—
Yes	5 (11)	For example, only pregnant patients
Target population (No of patients)		
Patients who underwent surgery	43 (91)	—
Patients who did or did not undergo surgery	4 (9)	—

ADNEX, Assessment of Different Neoplasias in the adnexa; CA125, cancer antigen 125.

ADNEX with CA125 was validated in 37 (79%) studies, and ADNEX without CA125 in 19 (40%) studies (16 studies evaluated both versions). Three (6%) studies conducted a mixed validation; ADNEX with CA125 was used when CA125 was available. For four (9%) studies, the ADNEX version was unclear. In total, 63 validations of ADNEX were performed after distinguishing between the ADNEX versions used (online supplemental table S4). When reported results for subgroups (eg, by menopausal status or by centre in multicentre studies) were also included, the total number of validations reported for the 47 studies was 159.

Five (11%) studies focused only on a specific clinical subgroup, such as pregnant patients or tumours, and the clinician's subjective assessment of the outcome was uncertain.^9 47 61 65 75^ Eight (17%) studies selected patients based on histology (online supplemental table S3). Thirty six (77%) studies did not focus on a specific clinical subgroup and did not select tumours based on histology. In these 36 studies that were eligible for the meta-analysis, median sample size was 284 tumours (range 50-4905), median number of malignant tumours was 68 (7-1041), and median prevalence of malignancy was 28% (3–57%). Fourteen of the 36 (39%) studies had ≥100 benign and ≥100 malignant tumours.

The target population of the studies was patients who were managed with surgery, or patients who were managed surgically and non-surgically. The reference standard for determining the type of tumour in patients who underwent surgery was always histopathology. Four (9%) studies included patients who were managed with and without surgery. In patients who did not undergo surgery, the outcome determination was the clinician's subjective assessment of the tumour as benign or malignant, or spontaneous resolution of the tumour during follow-up. The required follow-up time to determine the outcome was 3-4 months, one year, or two years, depending on the study.^6–9^

The most commonly reported performance measure was AUC for benign versus malignant tumours (72%) (online supplemental table S5). About two thirds of studies (66%) presented a receiver operating characteristic curve, 31 (66%) reported sensitivity and specificity performance at the 10% threshold for the risk of malignancy, 12 (26%) reported measures for multinomial discrimination, and four (9%) studies reported calibration performance.

### Critical appraisal: reporting completeness and risk of bias

**Figure 2:**
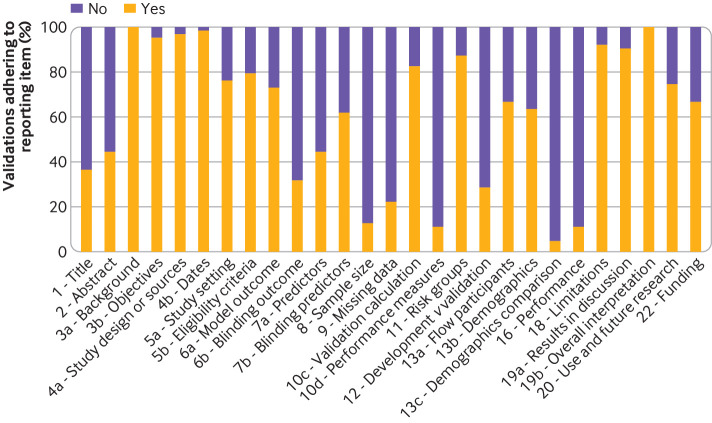
Adherence to reporting the TRIPOD (Transparent Reporting of a multivariable prediction model for Individual Prognosis Or Diagnosis) items in 63 validations

Completeness of reporting the TRIPOD items was assessed for the 63 validations. Adherence to TRIPOD items was, on average, 61%: studies reported a mean of 16.5 out of 27 items (figure 2 and online supplemental figure S1). The least commonly reported items were comparison of demographics, predictors, and outcome between the model development and external validation data (item 13c; 5%), reporting of performance measures with confidence intervals (item 16; 11%), specification of all performance measures (item 10d; 11%), rationale for study sample size (item 8; 13%), and description of how missing data were handled (item 9; 22%).

**Figure 3:**
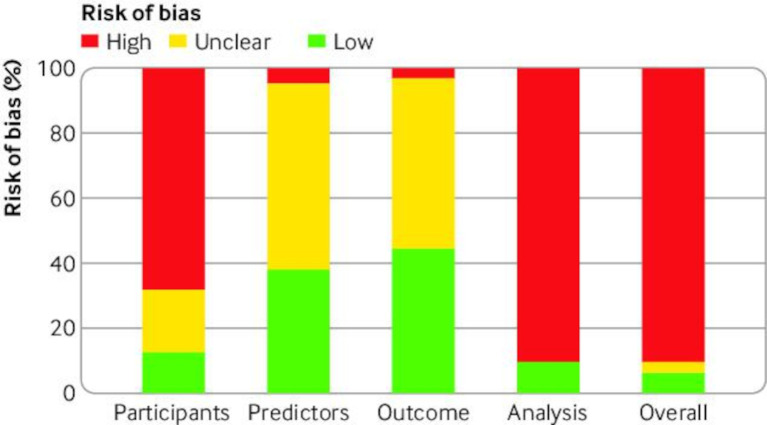
Risk of bias overall and by subdomain in 63 validations, assessed by PROBAST (Prediction model Risk Of Bias Assessment Tool)

Fifty seven (90%) of 63 validations were rated as having a high risk of bias, two (3%) as uncertain risk of bias, and four (6%) as low risk of bias (figure 3, online supplemental figures S2-5, and Open Science Framework repository, extraction sheet https://osf.io/jtsvd/).^29^ Forty three (68%) validations had a high risk of bias for the participant domain, mostly by having incomplete data as an exclusion criterion. Fifty seven (90%) validations had a high risk of bias for the analysis domain, mostly because of small sample size (69%; ie, <100 tumours in the smallest group), not including all participants in the analysis (85%), inappropriate handling of missing data (82%), and incomplete evaluation of model performance (89%, in most instances by not reporting an assessment of calibration).

Adherence to TRIPOD items in 36 studies without a focus on selected histologies or clinical subgroups was, on average, 65% (17.47 of 27 items). In these studies, two had a low risk of bias, one had an unclear risk of bias, and 33 had a high risk of bias.

### Meta-analyses of performance

**Figure 4:**
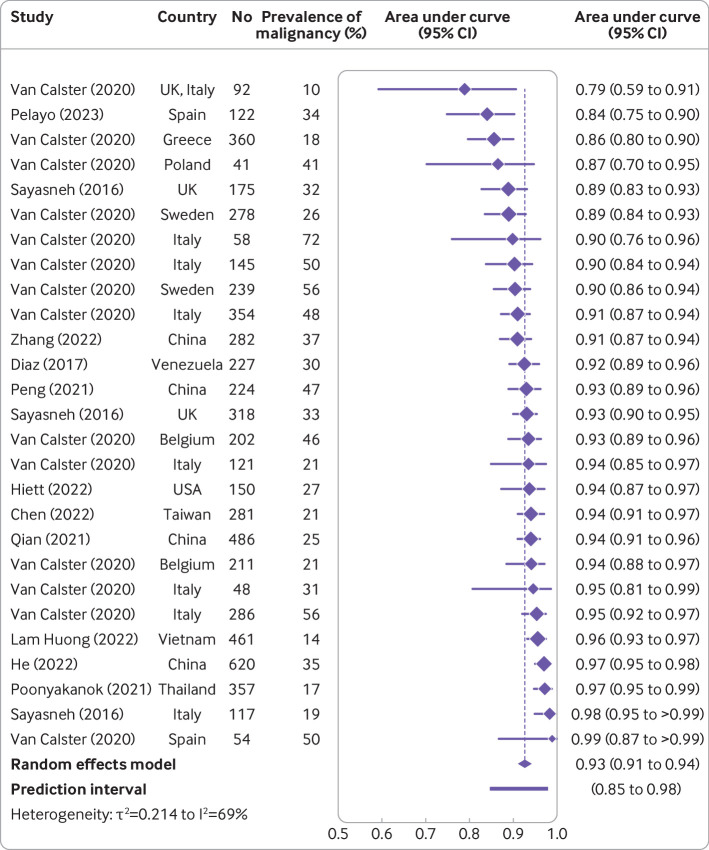
Forest plot of area under the receiver operating curve in studies where the ADNEX (Assessment of Different Neoplasias in the adnexa) model was used without CA125 (cancer antigen 125).^6 45 49 53 54 60 66–69 72 83^ Results in the forest plot are centre specific results, so studies with more than one centre can appear multiple times. CI=confidence interval

**Figure 5:**
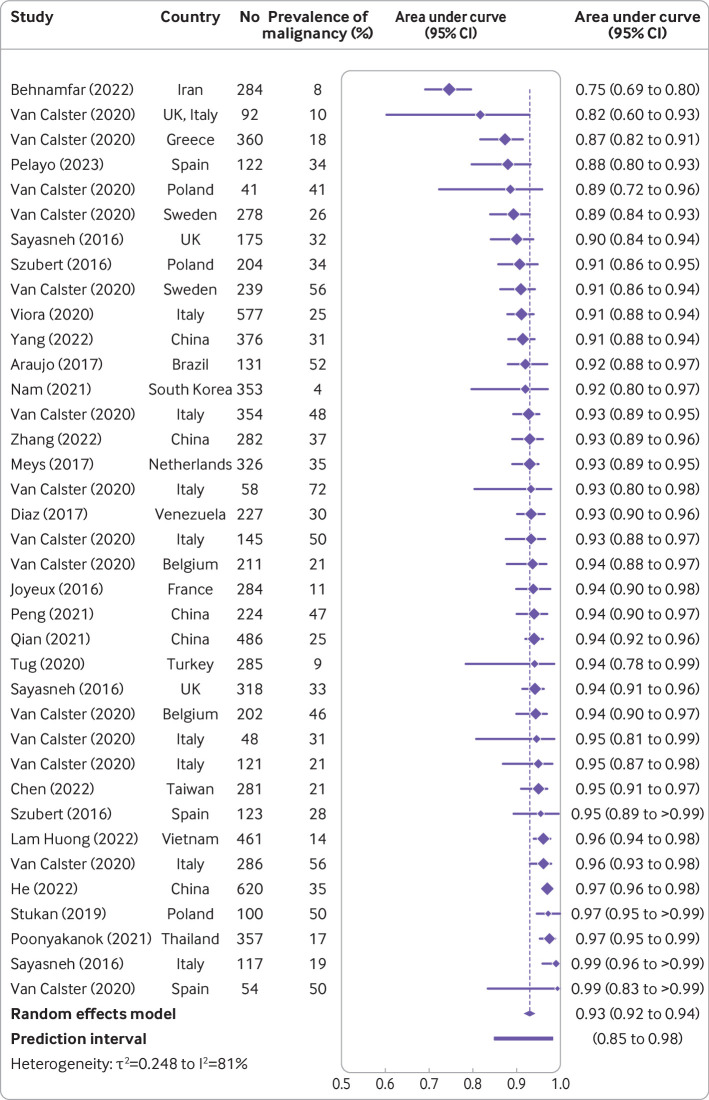
Forest plot of area under the receiver operating characteristic curve in studies where the ADNEX (Assessment of Different Neoplasias in the adnexa) model was used with CA125 (cancer antigen 125).^6 41 42 46 49 53 58 60 63 64 66–69 72 74 76 78 80 82 83^ Results in the forest plot are centre specific results, so studies with more than one centre can appear multiple times. CI=confidence interval

The meta-analysis included studies without post hoc selection based on histology and without a focus on a clinical subgroup only (n=36) that reported the meta-analysed metrics. Only two studies included in the meta-analysis used tumour as the unit of study.^42 51^ The AUC for benign versus malignant tumours in patients who underwent surgery was reported in 12 studies (6309 tumours, 31 centres, and 13 countries) for ADNEX without CA125 (online supplemental table S4 and table S6), and the summary AUC was 0.93 (95% confidence interval 0.91 to 0.94, 95% prediction interval 0.85 to 0.98) (table 2 and figure 4).^6 45 49 53 54 60 66–69 72 83^ Twenty one studies (9202 tumours, 43 centres, and 18 countries) reported the AUC for benign versus malignant tumours in patients who underwent surgery for ADNEX with CA125^6 41 42 46 49 53 58 60 63 64 67–69 72 74–76 78 80 82 83^ (online supplemental table S4 and table S6) and the summary AUC was 0.93 (95% confidence interval 0.92 to 0.94, 95% prediction interval 0.85 to 0.98) (table 2 and figure 5).

**Table 2:** Meta-analysis of area under the receiver operating characteristic curve to differentiate between benign and malignant tumours, including sensitivity and subgroup analyses

Meta-analysis	No of studies (centres)	Summary estimate (95% CI)	95% PI	τ^2^
Main analysis				
Patients who underwent surgery, with CA125	21 (43)	0.93 (0.92 to 0.94)	0.85 to 0.98	0.25
Patients who underwent surgery, without CA125	12 (31)	0.93 (0.91 to 0.94)	0.85 to 0.98	0.21
Sensitivity analyses (operated patients)				
High or unclear risk of bias studies, with CA125	19 (23)	0.93 (0.91 to 0.95)	0.84 to 0.99	0.33
Low risk of bias studies, with CA125	2 (20)	0.93 (0.91 to 0.94)	0.87 to 0.97	0.12
IOTA studies, with CA125	4 (23)	0.92 (0.91 to 0.94)	0.88 to 0.97	0.09
Non-IOTA studies, with CA125	17 (20)	0.93 (0.91 to 0.95)	0.83 to 0.99	0.38
High or unclear risk of bias studies, without CA125	10 (11)	0.94 (0.92 to 0.96)	0.86 to 0.99	0.26
Low risk of bias studies, without CA125	2 (20)	0.91 (0.90 to 0.93)	0.85 to 0.96	0.11
IOTA studies, without CA125	2 (20)	0.91 (0.89 to 0.93)	0.85 to 0.97	0.14
Non-IOTA studies, without CA125	10 (11)	0.94 (0.92 to 0.96)	0.86 to 0.99	0.26
Subgroup analyses (patients who underwent surgery)				
Asian centres, with CA125	11 (13)	0.94 (0.91 to 0.96)	0.81 to >.99	0.54
Asian centres, without CA125	7 (8)	0.95 (0.93 to 0.97)	0.89 to 0.99	0.19
Chinese centres, with CA125	5 (5)	0.94 (0.91 to 0.97)	0.87 to 0.99	0.25
Chinese centres, without CA125	4 (4)	0.94 (0.90 to 0.97)	0.85 to 0.99	0.33
European centres, with CA125	8 (28)	0.92 (0.91 to 0.94)	0.88 to 0.96	0.07
European centres, without CA125	3 (21)	0.91 (0.89 to 0.93)	0.84 to 0.97	0.16
Non-oncology centres, with CA125	2 (9)	0.91 (0.88 to 0.94)	0.85 to 0.97	0.11
Non-oncology centres, without CA125	1 (8)	0.90 (0.86 to 0.95)	0.80 to 0.99	0.24
Oncology centres, with CA125	18 (31)	0.93 (0.92 to 0.95)	0.84 to 0.98	0.29
Oncology centres, without CA125	12 (23)	0.93 (0.91 to 0.94)	0.85 to 0.98	0.22
Postmenopausal patients, with CA125	11 (32)	0.92 (0.90 to 0.94)	0.83 to 0.98	0.25
Postmenopausal patients, without CA125	4 (22)	0.90 (0.87 to 0.93)	0.81 to 0.97	0.19
Premenopausal patients, with CA125	11 (32)	0.91 (0.89 to 0.94)	0.81 to 0.98	0.28
Premenopausal patients, without CA125	4 (22)	0.91 (0.89 to 0.93)	0.85 to 0.96	0.08
Target population				
Patients managed with and without surgery, with CA125	2 (18)	0.94 (0.93 to 0.96)	0.88 to 0.99	0.22
Patients managed with and without surgery, without CA125	1 (17)	0.94 (0.91 to 0.95)	0.82 to 0.98	0.27

*Follow up time was 10-14 months (one year) or two years.

ADNEX, Assessment of Different NEoplasias in the adneXa; CA125, cancer antigen 125; CI, confidence interval; IOTA, International Ovarian Tumour Analysis; PI, prediction interval.

**Figure 6:**
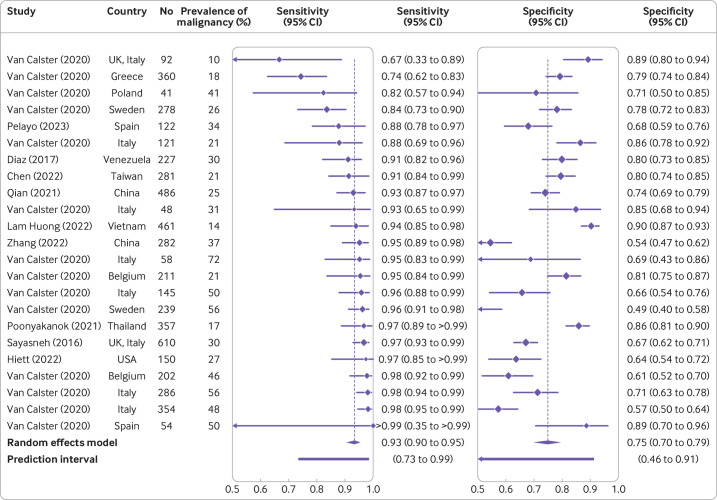
Forest plot of sensitivity and specificity at the 10% risk of malignancy threshold in studies where the ADNEX (Assessment of Different Neoplasias in the adnexa) model was used without CA125 (cancer antigen 125).^6 45 49 54 60 66 68 69 72 83^ Results in the forest plot are centre specific results, so studies with more than one centre can appear multiple times. CI=confidence interval

**Figure 7:**
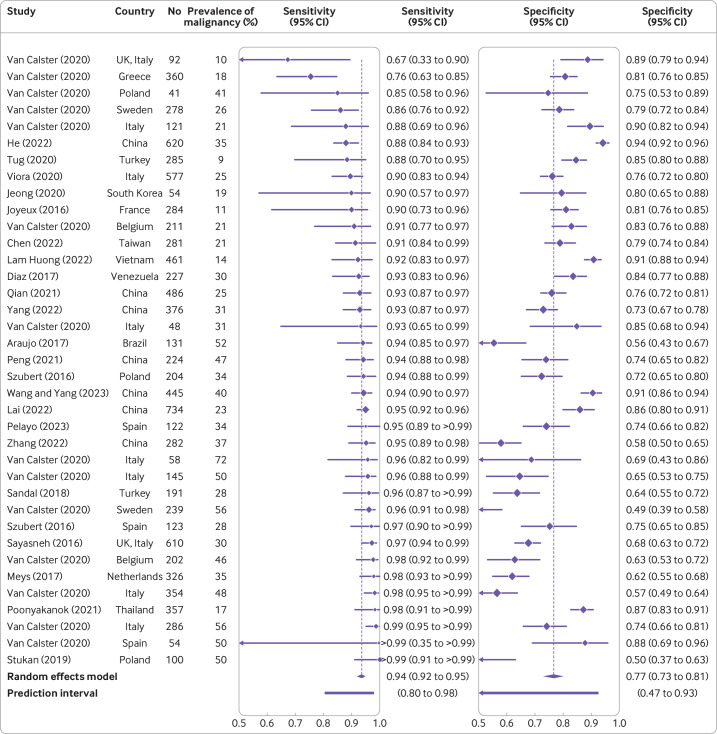
Forest plot of sensitivity and specificity at the 10% risk of malignancy threshold in studies where the ADNEX (Assessment of Different Neoplasias in the adnexa) model was used with CA125 (cancer antigen 125).^6 8 41 45 49 53 58–60 63 66–69 71 72 74 76 78 80–83^ Results in the forest plot are centre specific results, so studies with more than one centre can appear multiple times. CI=confidence interval

Sensitivity and specificity at the 10% risk of malignancy threshold in patients who underwent surgery were reported in 10 studies for ADNEX without CA125 (online supplemental table S4 and table S7).^6 45 49 54 60 66 68 69 72 83^ The summary sensitivity and specificity were 0.93 (95% confidence interval 0.90 to 0.95, 95% prediction interval 0.73 to 0.99) and 0.75 (95% confidence interval 0.70 to 0.79, 95% prediction interval 0.46 to 0.91), respectively (figure 6 and online supplemental table S8). For ADNEX with CA125, sensitivity and specificity at the 10% risk of malignancy threshold in patients who underwent surgery were reported in 23 studies (online supplemental table S4 and table S7).^6 8 41 45 49 53 58–60 63 66–69 71 72 74 76 78 80–83^ The summary sensitivity and specificity were 0.94 (95% confidence interval 0.92 to 0.95, 95% prediction interval 0.80 to 0.98) and 0.77 (95% confidence interval 0.73 to 0.81, 95% prediction interval 0.47 to 0.93), respectively (figure 7 and online supplemental table S8).

Net benefit and relative utility were calculated based on studies that presented sensitivity and specificity at the 10% risk of malignancy threshold in patients who underwent surgery (online supplemental table S7). For ADNEX without CA125, the summary net benefit was 0.28 (95% confidence interval 0.21 to 0.35, 95% prediction interval 0.05 to 0.68) and the summary relative utility was 0.50 (95% confidence interval 0.37 to 0.62, 95% prediction interval −0.44 to 0.79). The probability that the model is clinically useful in a random new centre was estimated to be 91% (online supplemental table S9, figure S6, and figure S7). For ADNEX with CA125, the summary net benefit was 0.28 (95% confidence interval 0.22 to 0.33, 95% prediction interval 0.05 to 0.65), and the summary relative utility was 0.54 (95% confidence interval 0.45 to 0.61, 95% prediction interval −0.12 to 0.78). The probability that the model is clinically useful in a random new centre was estimated to be 95% (online supplemental table S9, figure S8,9).

Pairwise AUC values in patients who underwent surgery were reported in four studies for ADNEX without CA125 and in five studies for ADNEX with CA125 (online supplemental table S10).^6 68 72 80 83^ The summary pairwise AUC values for ADNEX without CA125 ranged from 0.66 (stage II-IV primary invasive *v* metastatic) to 0.97 (benign *v* stage II-IV primary invasive) (online supplemental table S11). For ADNEX with CA125, the summary pairwise AUC values ranged from 0.72 (borderline *v* stage I primary invasive) to 0.98 (benign *v* stage II-IV primary invasive).

The AUC for benign versus malignant tumours in patients managed with and without surgery combined was reported in two studies (5167 tumours, 18 centres, and eight countries) with a summary estimate of 0.94 (95% confidence interval 0.93 to 0.96, 95% prediction interval 0.88 to 0.99) for ADNEX with CA125 (table 2).^6 7^ ADNEX without CA125 was assessed in only one study (4905 tumours, 17 centres, and seven countries).^6^ This study reported a summary AUC of 0.94 (95% confidence interval 0.91 to 0.95, 95% prediction interval 0.82 to 0.98).

Table 2 and online supplemental tables S6-9 present the sensitivity and subgroup results for AUC, specificity, sensitivity, net benefit, and relative utility. These results showed that the findings were robust (AUC values ranged from 0.90 to 0.95 across all analyses) and clinical utility was suggested in all subgroups. When limiting the analyses to studies with a low risk of bias, summary AUC values were 0.93 (with CA125) and 0.91 (without CA125). Sensitivity was higher and specificity lower in oncology versus non-oncology centres and in patients who were postmenopausal versus premenopausal. In line with these findings, meta-regression suggested that the prevalence of malignancy was not related to AUC but was related positively to sensitivity and negatively to specificity (online supplemental figures S10-12).

Meta-analysis of calibration only in patients who underwent surgery was not feasible because only one study reported calibration slope and intercept.^6^ Four studies presented a calibration plot; in three studies^72 80 83^ the estimated risks were close to the observed risks and in one study^6^ the risk of malignancy was slightly underestimated (online supplemental material S8).

Based on the subdomains of the GRADE assessment, we found that the risk of bias in the studies included in this meta-analysis was a substantial limitation affecting the certainty of our meta-analysis results. Only two studies (representing 5511 tumours or 32% of the total tumours included in this review) were not classified as having a high risk of bias,^6 72^ but the sensitivity analysis according to risk of bias showed consistent findings (table 2, and online supplemental table S8 and table S9). Funnel plots for AUC did not suggest publication bias (online supplemental figure S13).

## Discussion

### Principal findings

The ADNEX model performed well in classifying tumours and in differentiating between benign and malignant tumours across various settings and populations. Our results indicated that ADNEX was clinically useful at the 10% risk of malignancy threshold (eg, to help decide whether a patient should be referred for assessment to a gynaecological oncology centre). We found deficiencies in study reporting, and most studies were judged to have a high risk of bias, but our sensitivity analyses indicated that performance was almost identical in studies with a low risk and high risk of bias.

For ADNEX with CA125, the AUC was 0.93 based on all studies, versus 0.93 when based only on studies with a low risk of bias. For ADNEX without CA125, the AUC values were 0.93 (all studies) and 0.91 (low risk of bias only). High risk of bias was mainly caused by small sample size, no assessment of calibration performance, and unjustified use of complete case analysis. Small sample size implies that the estimated AUC value is less precise but it does not systematically affect the AUC, unless publication bias exists. The funnel plots did not suggest publication bias. Absence of calibration does not affect the AUC. Using complete cases, in terms of CA125 or other predictors, might lead to underestimation of AUC because missing values tend to be associated with the examiner's subjective impression that the tumour is benign.^84^ Complete case analysis would then tend to exclude clearly benign tumours, which would make the sample more homogeneous and reduce the AUC. Our results, however, suggest that the effect of complete case analysis on performance might have been minimal. The effect on calibration could not be assessed. Taken together, we believe that the results of our meta-analysis are reliable.

### Strengths and limitations of this study

Strengths of our systematic review include meta-analysis of ADNEX both as a risk model and as a diagnostic test, and the thorough critical appraisal of risk of bias and reporting quality with recommended checklists.^21 28^ Our study also had limitations. Some of the authors of this study had a conflict of interest because of their involvement in developing ADNEX or in some of the included external validation studies. To deal with this conflict of interest, independent researchers with expertise in study methodology and prediction modelling evaluated the IOTA related studies. A limitation of our findings (but not of our study) is that calibration performance was reported in only four studies and therefore meta-analysis of calibration was not possible.

### Comparison with other studies

Previous systematic reviews have conducted meta-analyses of the diagnostic performance of ADNEX with CA125.^14–18^ These studies used the QUADAS-2 (Quality Assessment of Diagnostic Accuracy Studies 2) tool^88^ to assess risk of bias, and found that 0-64% of studies had a high risk of bias in at least one domain. We identified 45 of 47 studies with a high risk of bias with PROBAST, designed for the appraisal of risk prediction models. None of the previous meta-analyses of ADNEX included clinical utility, calibration, or AUC. Our results align with those of other systematic reviews of risk prediction modelling studies in various domains. These studies consistently indicated that reporting in the original studies was poor and that many studies had a high risk of bias.^89–95^ The results in terms of sensitivity and specificity of ADNEX in our meta-analysis, however, were similar to those in the other meta-analyses of ADNEX performance.

### Study implications

ADNEX is intended for use by gynaecologists to help them decide on the most appropriate management of an adnexal mass detected on ultrasound. Our findings support the use of ADNEX in choosing between surgery and conservative follow-up. Conservative follow-up might be appropriate in patients with a low risk of malignancy (eg, <1%, based on meta-analysis of patients managed with and without surgery). Our results also support the use of ADNEX in deciding on the most appropriate management if conservative management is not suitable (eg, surgery at a local hospital if the risk of malignancy is <10% or referral to an oncology centre if the risk is>10%; based on meta-analysis of patients who underwent surgery).^4^

ADNEX can also be helpful in deciding on the management of a suspected malignancy (ie, investigations to find the primary tumour if a metastasis in the ovary is likely, or fertility sparing surgery if a borderline tumour is likely; based on meta-analysis in patients who underwent surgery). Because the AUC values of ADNEX with and without CA125 were similar, and because adding CA125 mainly helps to distinguish between different types of malignant tumours, we argue that the main use of ADNEX without CA125 is to help decide whether conservative follow-up, surgery in a local centre, or referral to an oncology centre is appropriate. The main use of ADNEX with CA125 is to help decide on the optimal management of a tumour suspected to be malignant, because it differentiates better between malignant subtypes than ADNEX without CA125.

Although our findings suggest that ADNEX is clinically useful, well conducted validations of any model are always of value to monitor its performance in diverse regions and clinical settings, and over time.^96^ To improve the performance of ADNEX even more, efforts to update the ADNEX formula are of interest.^96 97^ If further validation studies are conducted, we recommend including a validation of ADNEX without CA125, using a sufficiently large sample that allows calibration and multinomial discrimination to be assessed, and including patients irrespective of whether they are managed surgically or non-surgically (despite the challenges about reference standard for patients managed without surgery).^34 98^ Methodological recommendations for validation studies include using available tools to guarantee adequate sample size, describe missing data in detail and use methods such as imputation when needed, and assess calibration performance.^24 97 99–101^ Adherence to the TRIPOD reporting checklists is important to maximise the value of the validation study (www.tripod-statement.org).

### Conclusions

ADNEX has been validated in many studies, with AUC values >0.90 in differentiating between benign and malignant tumours in various settings, and with strong results for its clinical utility at the 10% risk of malignancy threshold. Because of the lack of assessment of calibration in most studies, evaluating the accuracy of the estimated risks in a substantial way was not possible in this study.

## Data Availability

Data are available in a public, open access repository. All data relevant to the study are included in the article or uploaded as supplementary information. Some data were provided by the authors and were not public information; therefore this information was omitted from the public repository.
