# Supplementary material for: ADNEX risk prediction model for diagnosis of ovarian cancer: systematic review and meta-analysis of external validation studies
Source: BMJ Med. 2024 Feb 17;3(1):e000817. doi: 10.1136/bmjmed-2023-000817 (PMC10875560; doi:10.1136/bmjmed-2023-000817)
Supplement: Supplementary data [file bmjmed-2023-000817supp001.pdf]

The ADNEX model for ovarian cancer diagnosis:

A systematic review and meta-analysis of external validation studies

Lasai BARREÑADA, Ashleigh LEDGER, Paula DHIMAN, Gary S. COLLINS, Laure WYNANTS,

Jan Y. VERBAKEL, Dirk TIMMERMAN, Lil VALENTIN, Ben VAN CALSTER

SUPPLEMENTARY MATERIAL

Corresponding author

Ben Van Calster

KU Leuven, Department of Development and Regeneration

Herestraat 49 box 805

3000 Leuven

Belgium

Ben.vancalster@kuleuven.be

S1. ADNEX MODEL

The ADNEX model is a multinomial logistic regression model published in 2014.[1] It is based on data from 5909 patients recruited at 25 centres in Belgium, Sweden, Italy, Czech Republic, Poland, France, Spain, United Kingdom, China, and Canada. ADNEX estimates the risk of five types of tumour: benign, borderline, stage I primary ovarian malignancy, stage II-IV primary ovarian malignancy, and secondary metastatic malignancy. The model is based on nine clinical and ultrasound features: age of the patient (in years), serum CA125 (U/mL), maximum diameter of the lesion (in mm; 'mdl'), the proportion of solid tissue calculated as the maximum diameter of the largest solid component (in mm) divided by the maximum diameter of the lesion (value between 0 and 1; 'pst'), presence of more than 10 cyst locules (1 versus 0; 'tcl'), the number of papillary projections (0, 1, 2, 3, 4, with 4 indicating more than three; 'nps'), presence of acoustic shadows (1 versus 0; 'sha'), the presence of ascites (1 versus 0; 'asc'), and examination at an oncology centre (1 versus 0; 'oc'). The ultrasound measurements are performed in accordance with the IOTA 'terms and definitions' statement.[2] Centres were encouraged to measure the level of serum CA125 in all patients, but this was not a requirement for inclusion in the study. Measurement of CA125 was left to clinical judgment and local protocols. ADNEX is based on a multinomial logistic regression model with random intercepts for centre. The final formula sets the random intercepts to zero, and hence uses only the fixed intercepts. A version of ADNEX without CA125 was also developed, because CA125 is not always measured in clinical practice. The formula of **ADNEX with CA125** is:

$$\text{risk}_{\text{benign}} = \frac{1}{1+\exp(z_1)+\exp(z_2)+\exp(z_3)+\exp(z_4)}$$

$$\text{risk}_{\text{borderline}} = \frac{\exp(z_1)}{1+\exp(z_1)+\exp(z_2)+\exp(z_3)+\exp(z_4)}$$

$$\text{risk}_{\text{stage I cancer}} = \frac{\exp(z_2)}{1+\exp(z_1)+\exp(z_2)+\exp(z_3)+\exp(z_4)}$$

$$\text{risk}_{\text{stage II-IV cancer}} = \frac{\exp(z_3)}{1+\exp(z_1)+\exp(z_2)+\exp(z_3)+\exp(z_4)}$$

$$\text{risk}_{\text{secondary metastasis}} = \frac{\exp(z_4)}{1+\exp(z_1)+\exp(z_2)+\exp(z_3)+\exp(z_4)}$$

Where

$$z1 = -7.577663 + 0.004506 * age + 0.111642 * log2(ca125) + 0.372046 * log2(mdl) + 6.967853 * pst - 5.65588 * pst2 + 1.375079 * tcl + 0.604238 * nps - 2.04157 * sha + 0.971061 * asc + 0.953043 * onc$$

$$z2 = -12.276041 + 0.01726 * age + 0.197249 * log2(ca125) + 0.87353 * log2(mdl) + 9.583053 * pst - 5.83319 * pst2 + 0.791873 * tcl + 0.400369 * nps - 1.87763 * sha + 0.452731 * asc + 0.452484 * onc$$

$$z3 = -14.91583 + 0.051239 * age + 0.765456 * log2(ca125) + 0.430477 * log2(mdl) + 10.37696 * pst - 5.70975 * pst2 + 0.273692 * tcl + 0.389874 * nps - 2.35516 * sha + 1.348408 * asc + 0.459021 * onc$$

$$z4 = -11.909267 + 0.033601 * age + 0.276166 * log2(ca125) + 0.449025 * log2(mdl) + 6.644939 * pst - 2.3033 * pst2 + 0.89998 * tcl + 0.215645 * nps - 2.49845 * sha + 1.636407 * asc + 0.808887 * onc.$$

For **ADNEX without CA125**, use

$$z1 = -7.412534 + 0.003489 * age + 0.430701 * log2(mdl) + 7.117925 * pst - 5.74135 * pst2 + 1.343699 * tcl + 0.607211 * nps - 2.11885 * sha + 1.167767 * asc + 0.983227 * onc$$

$$z2 = -12.201607 + 0.017607 * age + 0.98728 * log2(mdl) + 10.07145 * pst - 6.17742 * pst2 + 0.763081 * tcl + 0.410449 * nps - 1.98073 * sha + 0.77054 * asc + 0.543677 * onc$$

$$z3 = -12.826207 + 0.045172 * age + 0.759002 * log2(mdl) + 11.83296 * pst - 6.64336 * pst2 + 0.316444 * tcl + 0.390959 * nps - 2.94082 * sha + 2.691276 * asc + 0.929483 * onc$$

$$z4 = -11.424379 + 0.033407 * age + 0.560396 * log2(mdl) + 7.264105 * pst - 2.77392 * pst2 + 0.983394 * tcl + 0.199164 * nps - 2.63702 * sha + 2.185574 * asc + 0.906249 * onc.$$

S2. SEARCH STRATEGY

Phase 1:

The following databases were searched for eligible studies:

Search string for **PubMed**: ADNEX [tiab] OR (assessment[tiab] AND "different neoplasias"[tiab] AND "adnexa"[tiab]) AND ("2014/10/18"[Date - Publication] : "3000"[Date - Publication])

Search string for **EMBASE:** ADNEX:ti,ab,kw OR (assessment:ti,ab,kw AND 'different neoplasias':ti,ab,kw AND 'adnexa':ti,ab,kw) AND [2014-2024]/py

Search string for **Web of Science Core Collection**: TS= ("ADNEX" OR ("assessment" AND "different neoplasias" AND "adnexa")) AND PY=(2014-2024)

Search string for **SCOPUS**: TITLE-ABS-KEY (“ADNEX" OR ("assessment" AND "different neoplasias" AND "adnexa")) AND PUBYEAR > 2013

Search string for **EuropePMC**: adnex AND (SRC:PPR)

Additionally all the citations of ADNEX original paper (https://pubmed.ncbi.nlm.nih.gov/25320247/) were retrieved in **PubMed, SCOPUS/EMBASE and Web of Science**. We also searched for studies in systematic reviews that mention the ADNEX model.

Phase 2:

For the included articles in phase 1, all the relevant citations and references not already checked in phase 1 were checked in **PubMed, SCOPUS/EMBASE and Web of Science** for inclusion assessment. To determine if a referenced paper or citation was relevant to the systematic review, the title and context in the included paper were used as recommended by Wohlin.[3] Phase 2 was performed at data extraction for included papers in phase 1.

The search was first conducted in 29th November 2022 and repeated in 3rd March 2023 , 15th May 2023 and 10th November 2023.

Two articles were written in a language that was not understood by any of the co-authors (Turkish and Indonesian). Because we did not find suitable translators, we used the automatic translation tool deepl.com.

S3. IOTA STEERING COMMITTEE

The members of the IOTA steering committee are:

Dirk Timmerman, MD PhD (founder and coordinator, gynaecologist, KU Leuven, Belgium)

Tom Bourne, MD PhD (founder, gynaecologist, Imperial College London, UK)

Lil Valentin, MD PhD (founder, gynaecologist, Lund University, Sweden)

Antonia Testa, MD PhD (gynaecologist, Università Cattolica del Sacro Cuore, Rome, Italy)

Wouter Froyman, MD PhD (gynaecologist, KU Leuven, Belgium)

Ben Van Calster, PhD (medical statistician, KU Leuven, Belgium)

S4. META-ANALYSIS METHODS FOR AUC, SENSITIVITY, SPECIFICITY

We first extracted all the performance metrics as they were reported in the studies. Some studies presented confidence intervals or standard errors for the metrics, others did not. We used the following methods to approximate the uncertainty.

Approximation of the standard error for the logit of the AUC was based on Newcombe's method 4 [4] implemented in the ccalc function from the "metamisc" R package [5]:

$$SE(logit\ c) \approx \frac{SE(c)}{c(1-c)} \approx \sqrt{\frac{1 + n^* \frac{1-c}{2-c} + \frac{(m^* c)}{1+c}}{mnc(1-c)}},$$

where c is the *c* statistic, *n* the number malignant tumours and *m* the number of benign tumours, and $n^* = m^* = \frac{1}{2}(m+n)$.

Approximation of standard error for sensitivity and specificity was based on Wilson's method [6,7] implemented in madad function from the mada R package [8]:

$$CI = \frac{\hat{p} + \frac{z^2}{2n} \pm z\sqrt{\frac{\hat{p}(1-\hat{p})}{n} + \frac{z^2}{4n^2}}}{1 + \frac{z^2}{n}},$$

with $\hat{p}$ as reported sensitivity/specificity, *n* the number of tumours, and z denotes the quantile of the standard normal distribution.

To obtain the summary estimates, we performed a random effects meta-analysis to account for differences between studies. Random effects weights were calculated with inverse variance method $w_k = \frac{1}{s_k^2 - \tau^2}$ with s_k^2 as the within study variance and τ^2 as the between study variance. We calculated τ^2 using Restricted Maximum Likelihood ("REML") [9].

Subgroup analysis was performed by selecting from the k studies the studies that were part of the subgroup and conducting the meta-analysis independently from the whole sample, hence we did not use a common τ^2. To assess the association of prevalence of malignancy with the AUC and sensitivity and specificity at the 10% risk of malignancy threshold, we used random effects meta-regression. This method is similar to a traditional regression but instead of patients as unit of analysis we have studies. Then the model takes into account between study heterogeneity and within study heterogeneity in the same way as meta-analysis. [10]

Confidence intervals of the random effects meta analysis are constructed using Sidik-Jonkman Hartung-Knapp method [11,12]:

$$Var_{HKSJ} = \frac{\sum w_k \left(\theta_i - \hat{\theta}\right)^2}{K - 1 \sum w_k}.$$

Prediction intervals for the AUC were calculated using Bayesian methods. We used weak priors based on half Student-t distribution with location m = 0, scale σ = 0.5 and ν degrees of freedom = 3 [5].

$$\tau_{discr} \sim\ Student(0, 0.5^2, 3)\ T\ [0, 10]$$

5

Prediction intervals for specificity and sensitivity were calculated under the assumption that the random effects of each study are normally distributed with between study standard deviation (τ) as follows:

$$PI = \hat{\mu} \pm t_{k-2}\sqrt{\hat{\tau} + SE(\hat{\mu})^2},$$

with $\hat{\mu}$ as the estimated pooled effect, t_{k-2} is the $100\left(1-\frac{\alpha}{2}\right)$ percentile of the t-student distribution with k-2 degrees of freedom, and k the number of studies in the meta-analysis.

S5. TRIVARIATE RANDOM EFFECTS META-ANALYSIS FOR NET BENEFIT

We conducted a random effects meta-analysis of Net Benefit (NB) of ADNEX at the 10% risk of malignancy threshold according to the methodology described in [13]. Relative Utility (RU) expresses NB as a percentage of the maximum possible utility: RU=1 indicates maximum possible utility, RU=0 means no utility, RU<0 means harm. No utility means that NB of ADNEX is not higher than NB of treating all patients (NB_{TA}) and NB of treating no one (NB_{TN}, which is 0 by definition). In that case, ADNEX is not better than simply assuming that everyone needs treatment or that no one needs treatment without the use of any model. Harm means that NB of ADNEX is lower than NB_{TA} or NB_{TN}. Harm means that you can make better decisions without the model.

NB, NB_{TA}, and RU are defined as follows:

$$NB = Se * P - w * (1 - Sp) * (1 - P),$$

$$NB_{TA} = P - w * (1 - P),$$

$$RU = \frac{NB - \max(0, NB_{TA})}{P - \max(0, NB_{TA})},$$

with Se sensitivity, P prevalence of malignancy, Sp specificity, and w the odds of the risk threshold. In our case, $w = 1/9$.

We used weak prior distributions for the sensitivity, specificity and prevalence based on the results reported in the ADNEX model development study (sensitivity 0.965, specificity 0.713, average centre-specific prevalence 0.330). We used normal priors with $Z \sim N(\log\left(\frac{0.965}{1-0.965}\right), \sqrt{1/0.05})$ $Z \sim N(\log\left(\frac{0.713}{1-0.713}\right), \sqrt{}(1/0.5))$, $Z \sim N(\log\left(\frac{0.330}{1-0.330}\right), \sqrt{}(1/0.5))$ for sensitivity, specificity and prevalence of malignancy, respectively. This assures prior probability distributions bounded by 0 and 1. All other priors were the default priors suggested by Wynants et al [13].

For Markov chain Monte Carlo (MCMC) sampling, we used 1000 samples per chain and a burn-in of 1000, and two chains. This was sufficient for convergence and to have MC error <5% of the standard deviation of the posterior distribution for all parameters of interest.

Note that we implemented the methodology described in (63) after considering a recent erratum [14]

S6. PUBLICATION BIAS

Publication bias and small study effects were explored with funnel plots for the AUC. We used random effects model to estimate the summary effect, and tau-squared was estimated using "REML" (Restricted maximum-likelihood estimator). The included studies were the same as those used in the meta-analysis of the AUC with CA125 (see Table S9, row 1).

The funnel plots should be interpreted with caution because deviations from the summary result can be due to publication bias or to differences in case-mix.

Barreñada L, *et al. BMJMED* 2024; 3:e000817. doi: 10.1136/bmjmed-2023-000817

S7. INCONSISTENCIES IN REPORTED DATA

Chen et al (2022) mentioned that there were 281 patients in Table 5 vs 279 in Supplementary Material. We opted for 281 because the calculated 2x2 table for specificity and sensitivity yielded numbers closer to integers.

Joyeux et al (2016) reported results for younger (up to 42 years) versus older (≥43 years) patients in Table 4. The number of patients in the older group was reported to be 188, but the authors sometimes reported that the younger group contained 96 patients and sometimes that it contained 97 patients. As the total sample size is 284, we decided that there were 96 younger patients.

Lam Huong (2022) reported in the abstract that there were 65 malignant and 361 benign tumours, but that the total sample size was 461. In the text and in tables, the authors reported on 396 benign tumours. We assumed that there was a typing error in the abstract and extracted 396 benign cases and 65 malignancies.

Nam et al (2021) reported a sensitivity of 0.90 with 13 malignancies which is not possible. It should be either 0.92 with 12 true positives or 0.84 with 11 true positives. For this reason, we decided to exclude this study from the meta-analysis of specificity and sensitivity.

Tavoraité et al (2021) reported a specificity of 0.46, but with the reported number of true negatives it should have been either 0.48 or 0.45. This study was not eligible for meta-analysis because they analysed ADNEX by the level of expertise of the examiners. According to the reported accuracy, we assumed the correct specificity to be 0.48 with 16 true negatives, 17 true positives, 17 false positives, and 0 false negatives.

Liu et al (2021) reported on sensitivity and specificity, yet only provided information on the number of malignant masses in the study. We decided to exclude this study from all numerical analysis, but we included the 84 malignancies reported by Liu et al in the total number of tumours in our systematic review.

Szubert et al (2020) reported on AUC and sensitivity and specificity for three subgroups that are stratified by the certainty of the subjective assessment: tumours for which the examiner states that it is ‘certainly benign’ or ‘certainly malignant’, tumours for which the examiner states it is ‘probably benign’ or ‘probably malignant’, and tumours for which the examiner is uncertain. However, for the group of certain tumours, the AUC and sensitivity were 1 but the specificity was not perfect. This is not possible, and we could not check the source of the inconsistency, because the authors did not report the number of cases in each subgroup. For this reason, we decided not to include this study’s metrics in our paper. This study was also not included in any meta-analysis, because no performance was reported for all tumours (irrespective of subjective assessment). This study is labelled as one that focuses on specific clinical subgroups, in this case subgroups depending on the subjective assessment of the examiner.

Barreñada L, *et al. BMJMED* 2024; 3:e000817. doi: 10.1136/bmjmed-2023-000817

S8. CALIBRATION PLOTS

Sayasneh et al. (2016) [15], © 2016 CC BY NC SA by Springer Nature

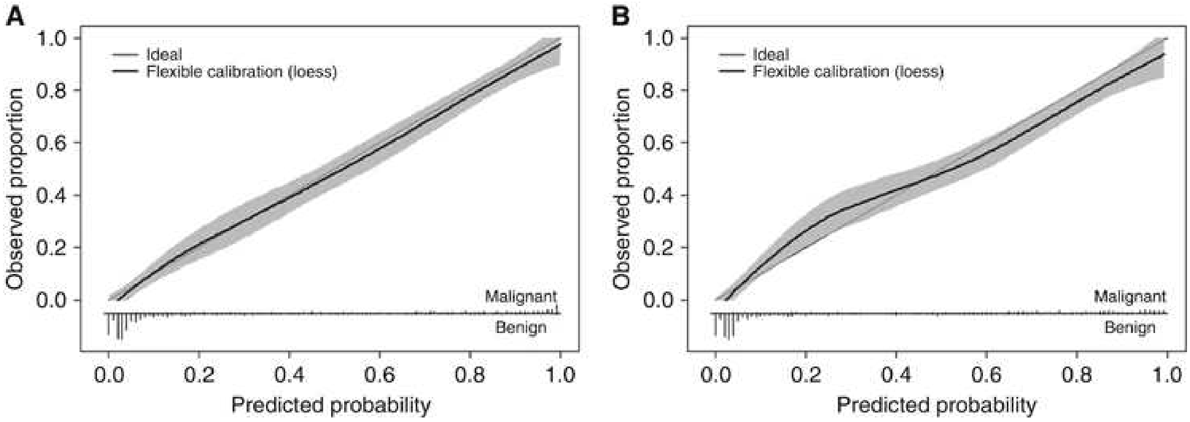

(A**) Calibration plot for the ADNEX model with serum CA125. (B) Calibration plot for the ADNEX model without serum CA125.**

Van Calster et al. (2020) [16], © 2020 CC BY by British Medical Journal Publishing Group

Model	Intercept (95% CI)	Slope (95% CI)
LR2	0.30 (-0.02 to 0.63)	0.84 (0.72 to 0.96)
SRRisk	0.14 (-0.19 to 0.47)	1.00 (0.87 to 1.13)
ADNEX without CA125	0.19 (-0.04 to 0.42)	1.12 (0.98 to 1.26)
ADNEX with CA125	0.19 (-0.01 to 0.40)	1.11 (0.98 to 1.25)

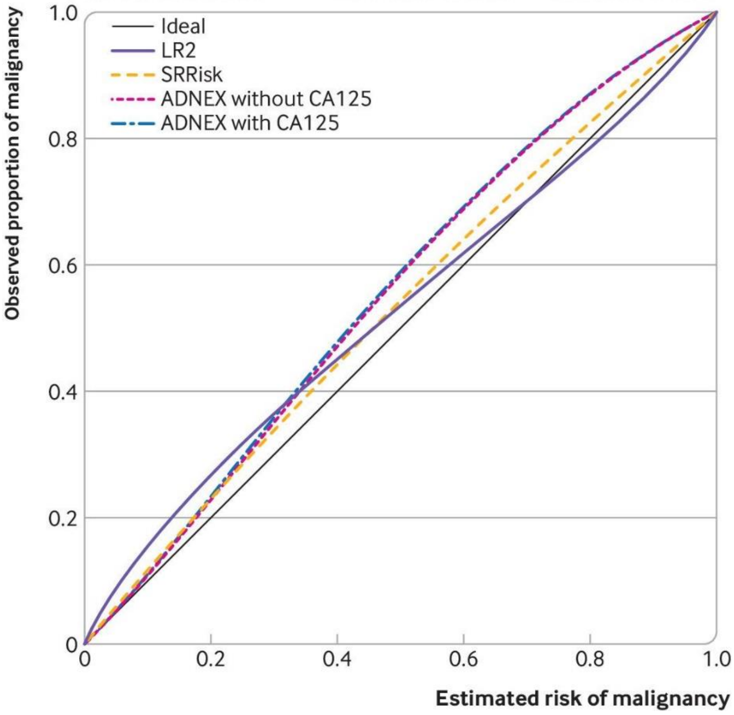

Summary figure with overall calibration curves for risk prediction models. ADNEX=assessment of different neoplasias in the adnexa; intercept=calibration intercept; LR2=logistic regression model 2; RMI=risk of malignancy index; slope=calibration slope; SRRisk=simple rules risk model

Viora et al. (2020) [17], reused for publication in BMJ Medicine with permission from Elsevier Publishing Group.

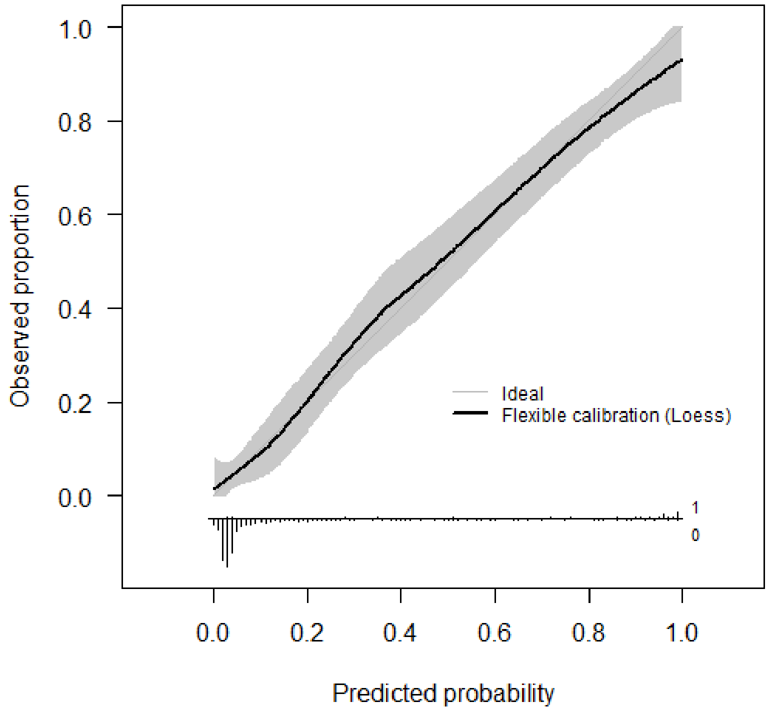

Calibration plot of predicted probability for the total risk of malignancy.

Zhang et al. (2022)[18] , reused for publication in BMJ Medicine with permission from Elsevier Publishing Group.

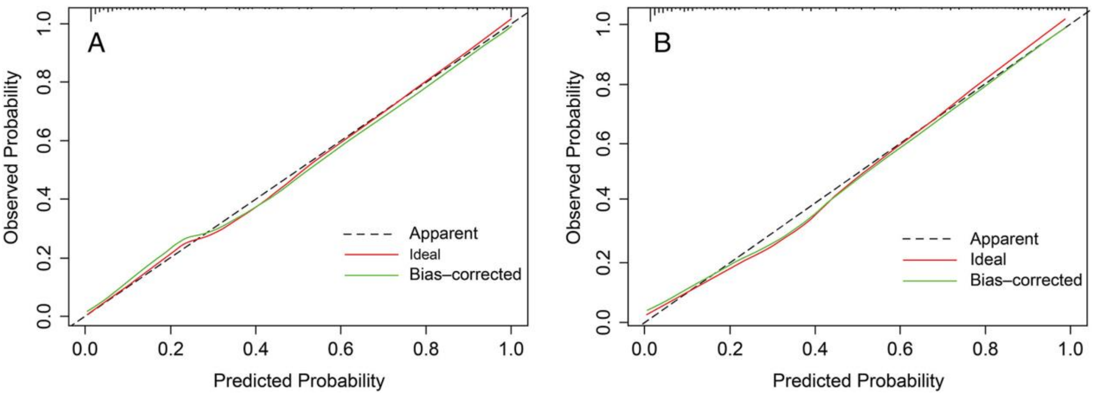

A, Calibration plot for the ADNEX model with CA125. B, Calibration plot for the ADNEX model without CA125. ADNEX, Assessment of Different NEoplasias in the adneXa.

SUPPLEMENTARY TABLES

Table S1. Data extracted from each validation study.

	Item	Values
**General information**	Name of the reviewer	Name
	Number of validations (e.g. total study population plus postmenopausal gives two validations)	Number of validations
	Unit of study	Patient or tumour
	Version of ADNEX	With CA125, without CA125, unclear
**Target population and setting**	Single country	Yes or No
	Number of countries	Number of countries
	Start date and end date of recruitment	Start and end date
	Target population	Operated only, or both operated and managed with follow-up
**Study description**	Study design	Prospective, retrospective, ambispective, unclear cohort.
	Setting	Oncology, Non-oncology, unclear
	Recruitment method	Consecutive, probably consecutive, other, unclear
	Number of centres	Monocentric or multicentric and number of centres
	Inclusion criteria & Exclusion criteria	Listed as in the study
	Missing data as exclusion criteria	Yes, No, unclear
	Exclusion variables	ADNEX predictors or outcome with missing data that resulted in exclusion of the patient or tumour
	Number of excluded patients because of missing data	Number of excluded patients
	How are borderline ovarian tumours treated	Benign, malignant, other
**Predictors**	Measurement used in the study	Mean, Median, Unclear, Not reported
	Variability measure	Standard deviation, Interquartile range, Range, Unclear, Not reported
	Age of the population	Age (Variability)
	Reported descriptive statistics for the ADNEX predictors	Age; CA125; Family history; Maximal diameter; Solid tissue; Papillary projections; >10 cyst locules; Shadows; Ascites
	Reported descriptive statistics of predictors by outcome	Total; Benign; Borderline; Stage I; Stage II-IV; Metastasic; Malignant
**Subgroup analysis**	Menopausal data	Yes or No
	Conservative follow-up	Yes or No; if yes, duration of follow up
**Outcome**	Type	Multinomial or binary
	Reference standard	Histology, Other
	Sample size and number of malignancies and tumour subtypes	Number
	Malignancy rate	Percentage
**Analysis**	Missing data	Number of patients with missing information for any variable
	Handling of missing data	Complete case analysis, single imputation, multiple imputation, not reported
	Software for missing data	Python, R, Stata, SAS, Not reported
	AUC as the sum of two triangles	Yes or no
	Performance AUC Benign vs Malignant	AUC (CI 95%)
	AUC ROC plot	Yes or No
	Pairwise AUC methodology	Conditional risk, other, not reported
	Performance pairwise AUC	AUC (CI 95%) for the 10 possible pairs
	Performance PDI	PDI
	Calibration plot	Yes or no
	Calibration intercept and slope	Calibration slope and intercept
	Multinomial calibration plot	Yes or No
	Risk of malignancy cut-off	Cut-off
	Sensitivity/Specificity at all cut-offs	Sensitivity, specificity (CI 95%)
	PPV and NPV at 10%	PPV, NPV (CI 95%)
	DOR at 10%	DOR
	Net benefit	Net benefit
	Extra reported metrics	Names of metrics
**General information**	Statistical software used	Name of software(s)
	Conclusion or general opinion on ADNEX	Text
**TRIPOD**	All applicable tripod items (See Table S3)	Yes or No, if NO with an explanation for our classifying an item as not having been addressed by the authors
**PROBAST**	All applicable signalling questions and risk of bias (ROB) assessment by subdomain and overall ROB	Yes, Probably Yes, No, Probably no, No information for signalling questions.<br>Low, Unclear, High ROB<br>Arguments for ROB classification when needed

AUC, area under the receiver operating characteristic curve; ROC, receiver operating characteristic curve; PDI, polytomous discrimination index; PPV, positive predictive value; NPV, negative predictive value; DOR, diagnostic odds ratio; CI, confidence interval.

Table S2. Transparent reporting of a multivariable prediction model for individual prognosis or diagnosis (TRIPOD) items.

Section/Topic	Item	Checklist Item
Title	1	Identify the study as developing and/or validating a multivariable prediction model, the target population, and the outcome to be predicted.
Abstract	2	Provide a summary of objectives, study design, setting, participants, sample size, predictors, outcome, statistical analysis, results, and conclusions.
Background and objectives	3a	Explain the medical context (including whether diagnostic or prognostic) and rationale for developing or validating the multivariable prediction model, including references to existing models.
	3b	Specify the objectives, including whether the study describes the development or validation of the model or both.
Source of data	4a	Describe the study design or source of data (e.g., randomized trial, cohort, or registry data), separately for the development and validation data sets, if applicable.
	4b	Specify the key study dates, including start of accrual; end of accrual; and, if applicable, end of follow-up.
Participants	5a	Specify key elements of the study setting (e.g., primary care, secondary care, general population) including number and location of centres.
	5b	Describe eligibility criteria for participants.
	5c	Give details of treatments received, if relevant.
Outcome	6a	Clearly define the outcome that is predicted by the prediction model, including how and when assessed.
	6b	Report any actions to blind assessment of the outcome to be predicted.
Predictors	7a	Clearly define all predictors used in developing or validating the multivariable prediction model, including how and when they were measured.
	7b	Report any actions to blind assessment of predictors for the outcome and other predictors.
Sample size	8	Explain how the study size was arrived at.
Missing data	9	Describe how missing data were handled (e.g., complete-case analysis, single imputation, multiple imputation) with details of any imputation method.
Statistical analysis methods	10c	For validation, describe how the predictions were calculated.
	10d	Specify all measures used to assess model performance and, if relevant, to compare multiple models.
Risk groups	11	Provide details on how risk groups were created, if done.
Development vs. validation	12	For validation, identify any differences from the development data in setting, eligibility criteria, outcome, and predictors.
Participants	13a	Describe the flow of participants through the study, including the number of participants with and without the outcome and, if applicable, a summary of the follow-up time. A diagram may be helpful.
	13b	Describe the characteristics of the participants (basic demographics, clinical features, available predictors), including the number of participants with missing data for predictors and outcome.
	13c	For validation, show a comparison with the development data of the distribution of important variables (demographics, predictors and outcome).
Model performance	16	Report performance measures (with CIs) for the prediction model.
Limitations	18	Discuss any limitations of the study (such as nonrepresentative sample, few events per predictor, missing data).
Interpretation	19a	For validation, discuss the results with reference to performance in the development data, and any other validation data.
	19b	Give an overall interpretation of the results, considering objectives, limitations, results from similar studies, and other relevant evidence.
Implications	20	Discuss the potential clinical use of the model and implications for future research.
Supplementary information	21	Provide information about the availability of supplementary resources, such as study protocol, Web calculator, and data sets.
Funding	22	Give the source of funding and the role of the funders for the present study.

For more information see [19,20]

Table S3. Descriptive characteristics of included studies (n=47).

Study	Region	Type of centre	Number of centres	Unit	Clin/Histo Focus[1]	N (benign - malignant)	ADNEX version
**Epstein (2016) [21]** [2]	Europe	Oncology	1	Patient	Yes	126 (0-126)	With CA125
**Joyeux (2016) [22]**	Europe	Unclear	2	Patient	No	284 (254-30)	With CA125
**Sayasneh (2016) [15]** [2]	Europe	Oncology	3	Patient	No	610 (428-182)	Both
**Szubert (2016) [23]** [2]	Europe	Oncology	1	Patient	No	204 (134-70)	Mixed[3]
**Araujo (2017) [24]**	South America	Oncology	1	Patient	No	131 (63-68)	With CA125
**Diaz (2017) [25]**	South America	Oncology	1	Patient	No	227 (159-68)	Both
**Meys (2017) [26]** [2]	Europe	Oncology	1	Patient	No	326 (211-115)	With CA125
**Sandal (2018) [27]**	Asia	Oncology	1	Patient	No	191 (138-53)	With CA125
**Chen (2019) [28]**	Asia	Oncology	1	Patient	No	278 (203-75)	Both
**Nohuz (2019) [29]**	Europe	Oncology	1	Patient	Yes	93 (89-4)	With CA125
**Stukan, Alcazar (2019) [30]** [2]	Europe	Oncology	7	Patient	Yes	162 (0-162)	Both
**Stukan, Badocha (2019) [31]**	Europe	Oncology	1	Patient	No	100 (50-50)	With CA125
**Gaurilcikas (2020) [32]**	Europe	Oncology	1	Patient	Yes	85 (0-85)	Both
**Jeong (2020) [33]**	Asia	Oncology	1	Patient	No	59 (49-10)	With CA125
**Quaranta (2020) [34]**	Europe	Oncology	1	Patient	Yes	34 (34-0)	With CA125
**Szubert (2020) [35]**	Europe	Oncology	2	Tumor	Yes	451 (250-201)	With CA125
**Tug (2020) [36]**	Asia	Unclear	1	Patient	No	285 (259-26)	With CA125
**Van Calster (2020) [16]** [2]	Europe	Both	17	Patient	No	4905 (3864-1041)	Both
**Viora (2020) [17]**	Europe	Non-oncology	1	Patient	No	577 (433-144)	With CA125
**Butureanu (2021) [37]**	Europe	Unclear	1	Patient	No	230 (223-7)	With CA125
**Czekierdowski (2021) [38]**	Europe	Both	4	Patient	Yes	36 (27-9)	Without CA125
**Jiang (2021) [39]**	Asia	Oncology	1	Patient	Yes	63 (42-21)	With CA125
**Lee (2021) [40]**	Asia	Oncology	11	Patient	Yes	236 (223-13)	Unclear
**Liu (2021) [41]**	Asia	Unclear	1	Patient	No	Unclear (Unclear-84)	Unclear
**Nam (2021) [42]**	Asia	Oncology	1	Patient	No	353 (340-13)	With CA125
**Peng (2021) [43]**	Asia	Oncology	1	Patient	No	224 (119-105)	Both
**Poonyakanok (2021) [44]**	Asia	Oncology	1	Patient	No	357 (296-61)	Both
**Qian (2021) [45]**	Asia	Oncology	1	Patient	No	486 (366-120)	Both
**Tavoraitè (2021) [46]**	Europe	Oncology	1	Patient	No	50 (33-17)	Mixed[3]
**Behnamfar (2022) [47]**	Asia	Oncology	2	Tumor	No	284 (260-24)	With CA125
**Budiana (2022) [48]**	Asia	Oncology	1	Tumor	No	88 (38-50)	Unclear
**Chen (2022) [49]**	Asia	Oncology	1	Patient	No	322 (264-58)	Both
**Esquivel Villabona (2022) [50]**	South America	Oncology	1	Tumor	No	606 (545-61)	Unclear
**Hack (2022) [51]**	North America	Oncology	1	Tumor	No	262 (187-75)	Mixed[3]
**He (2021) [52]**	Asia	Oncology	1	Patient	No	620 (402-218)	Both
**Hiett (2022) [53]**	North America	Oncology	1	Patient	No	150 (110-40)	Without CA125
**Jianhong (2022) [54]**	Asia	Oncology	1	Patient	Yes	23 (15-8)	Both
**Lai (2022) [55]**	Asia	Unclear	1	Patient	No	734 (564-170)	With CA125
**Lam Huong (2022) [56]**	Asia	Oncology	2	Patient	No	461 (396-65)	Both
**Velayo (2022) [57]**	Asia	Oncology	2	Patient	No	260 (141-119)	With CA125
**Yang (2022) [58]**	Asia	Oncology	1	Patient	No	376 (259-117)	With CA125
**Zhang (2022) [18]**	Asia	Oncology	1	Patient	No	282 (178-104)	Both
**Czekierdowski (2023) [59]**	Europe	Both	3	Patient	Yes	108 (62-46)	With CA125
**Hu (2023) [60]**	Asia	Oncology	2	Patient	No	529 (370-159)	Without CA125
**Pelayo (2023) [61]**	Europe	Oncology	1	Patient	No	122 (81-41)	Both
**Rashmi (2023) [62]**	Asia	Oncology	1	Patient	No	90 (80-10)	Both
**Wang And Yang (2023) [63]**	Asia	Unclear	1	Patient	No	445 (265-180)	With CA125

[1]A study was considered to have clinical or histological focus if the study sample consisted of selected histologies (e.g. only borderline tumours), or a selected subgroup of patients (e.g. only pregnant patients).

[2]Papers extracted by authors PD and GSC.

[3]Mixed" means that the authors used ADNEX with CA125 for patients with CA125 data and ADNEX without CA125 for patients without CA125 data.

Table S4. Reported performance for distinguishing benign from malignant tumours (63 validations)

Study	ADNEX With CA125	Missing Data handling	AUC Benign versus Malignant (95%CI)	Sensitivity at 10% cut-off (95% CI)	Specificity at 10% cut-off (95% CI)	Calibr.	RoB	TRIPOD items reported
**Epstein (2016)**	Yes	CCA	NR	NR	NR	No	High	61%
**Joyeux (2016)[a,b]**	Yes	CCA	0.938 (0.899-0.977)	90	81.1	No	High	64%
**Sayasneh (2016)[a,b]**	Yes	MI/SI	0.937 (0.915-0.954)	97.3 (93.5-98.9)	67.7 (63.0-72.0)	Yes	Low	86%
**Sayasneh (2016)[a,b]**	No	SI	0.925 (0.902-0.943)	96.7 (92.9-98.5)	67.1 (62.5-71.3)	Yes	Low	86%
**Szubert (2016)[a,b]**	Mixed	CCA	0.907 (0.858 - 0.948)	94.3 (88.5 - 98.7)	72.4 (65.1 - 79.7)	No	High	54%
**Araujo (2017)[a,b]**	Yes	CCA	0.92 (0.88-0.97)	94.1	55.5	No	High	68%
**Diaz (2017)[a,b]**	Yes	CCA	0.933 (0.901-0.964)	92.64	83.64	No	High	61%
**Diaz (2017)[a,b]**	No	CCA	0.925 (0.892-0.958)	91.17	79.87	No	High	64%
**Meys (2017) [b]**	Yes	MI	0.93 (0.89-0.95)	98 (93-100)	62 (55-68)	No	High	64%
**Sandal (2018)**	Yes	CCA	NR	96.2 (87 - 99.5)	63.7 (55.2- 71.7)	No	High	50%
**Chen (2019)**	Yes	CCA	0.94 (0.91-0.97)	93.3 (85-98)	77.8 (72-83)	No	High	68%
**Chen (2019)**	No	CCA	0.93 (0.90-0.96)	NR	NR	No	High	68%
**Nohuz (2019)**	Yes	CCA	NR	100	98.8	No	High	43%
**Stukan, Alcazar (2019)**	Yes	CCA	NR	NR	NR	No	High	61%
**Stukan, Alcazar (2019)**	No	CCA	NR	NR	NR	No	High	61%
**Stukan, Badocha (2019)[a,b]**	Yes	CCA	0.972 (0.946-0.999)	100	50	No	High	75%
**Gaurilcikas (2020)**	Yes	CCA	NR	NR	NR	No	High	54%
**Gaurilcikas (2020)**	No	CCA	NR	NR	NR	No	High	54%
**Jeong (2020)[b,c]**	Yes	NR	0.924 (0.786-1.0)	90	81.6	No	High	64%
**Quaranta (2020)**	Yes	CCA	NR	NR	NR	No	High	54%
**Szubert (2020)**	Yes	CCA	NR	NR	NR	No	High	54%
**Tug (2020)[a,b]**	Yes	CCA	0.941 (0.042)	88.5	84.6	No	High	64%
**Van Calster (2020)[a,b]**	Yes	MI	0.94 (0.92-0.96)	91.2 (84.8-95.1)	85.3 (80.9-88.8)	Yes	Low	100%
**Van Calster (2020)[a,b]**	No	MI	0.94 (0.91-0.95)	91.1 (84.5-95.1)	84.5 (80.1-88.0)	Yes	Low	100%
**Viora (2020)[a,b]**	Yes	CCA	0.9111 (0.8788-0.9389)	89.6 (83.1-94.0)	76.2 (71.9-80.1)	Yes	High	64%
**Butureanu (2021)**	Yes	NR	NR	NR	NR	No	High	43%
**Czekierdowski (2021)**	No	CCA	NR	NR	NR	No	High	64%
**Jiang (2021)**	Yes	NR	NR	NR	NR	No	High	43%
**Lee (2021)**	Unc	NR	0.709 (0.646-0.766)	NR	NR	No	High	50%
**Liu (2021) [c]**	Unc	NR	0.821	NR	NR	No	High	39%
**Nam (2021)[a,b]**	Yes	NR	0.92	90	82.0	No	High	61%
**Peng (2021)[a]**	Yes	NR	0.94 (0.90-0.97)	94.3 (88.0-97.9)	74.0 (65.1-81.6)	No	High	64%
**Peng (2021)[a,b]**	No	NR	0.93 (0.89-0.96)	NR	NR	No	High	68%
**Poonyakanok (2021)[a,b]**	Yes	CCA	0.975 (0.953-0.988)	98.4 (91.2-100)	87.2 (82.8-90.8)	No	High	75%
**Poonyakanok (2021)[a,b]**	No	CCA	0.972 (0.949-0.987)	96.7 (88.7-99.6)	85.8 (81.3-89.6)	No	High	79%
**Qian (2021)[a,b]**	Yes	CCA	0.94 (0.92-0.96)	93 (87-97)	76 (72-81)	No	High	68%
**Qian (2021)[a,b]**	No	CCA	0.94 (0.91-0.96)	93 (87-97)	74 (69-79)	No	High	68%
**Tavoraitè (2021)**	Mixed	CCA	NR	100 (80.5-100)	81.8 (64.5-93)	No	High	46%
**Behnamfar (2022)[a]**	Yes	NR	0.746 (0.691-0.796)	NR	NR	No	High	46%
**Budiana (2022)**	Unc	NR	NR	NR	NR	No	High	46%
**Chen (2022)[a]**	Yes	CCA	0.95 (0.91-0.97)	91.4 (84.2-98.6)	78.9 (73.6-84.3)	No	High	75%
**Chen (2022)[a]**	No	CCA	0.94 (0.91-0.97)	91.4 (84.2-98.6)	79.5 (74.2-84.6)	No	High	75%
**Esquivel Villabona (2022)[c]**	Unc	NR	0.895	91.8 (82.2-96.4)	87.2 (84.08-89.71)	No	High	64%
**Hack (2022)**	Mixed	CCA	0.9479	NR	NR	No	High	61%
**He (2022)[a]**	Yes	CCA	0.97 (0.96-0.98)	88.06 (83.58-92.54)	94.10 (91.79-96.41)	No	High	71%
**He (2022)[a,b]**	No	CCA	0.97 (0.95-0.98)	NR	NR	No	High	71%
**Hiett (2022)[a,b]**	No	NR	0.937	97.5 (85.3-99.9)	63.6 (53.9-72.4)	No	High	61%
**Jianhong (2022)**	Yes	CCA	0.892 (0.692-0.982)	87.50 (47.3-99.7)	73.33 (44.9-92.2)	No	High	57%
**Jianhong (2022)**	No	CCA	0.896 (0.697-0.983)	100.0 (63.1-100.0)	53.3 (26.6-78.7)	No	High	57%
**Lai (2022)[bc]**	Yes	CCA	0.90 (0.87-0.94)	95 (92-96)	86 (80-91)	No	High	57%
**Lam Huong (2022)[a,b]**	Yes	NR	0.961 (0.939 - 0.977)	92.3 (83.0 - 97.5)	90.9 (87.6 - 93.6)	No	High	43%
**Lam Huong (2022)[a,b]**	No	NR	0.956 (0.933 - 0.973)	93.9 (85.0 - 98.3)	90.2 (86.8 - 92.9)	No	High	46%
**Velayo (2022) [c]**	Yes	CCA	0.78 (0.73-0.82)	NR	NR	No	High	54%
**Yang (2022)[a,b]**	Yes	CCA	0.914 (0.881-0.941)	93 (87-97)	73 (67-78)	No	High	71%
**Zhang (2022)[a,b]**	Yes	SI	0.93 (0.89-0.96)	95.2 (89.1-98.4)	57.9 (50.3-65.2)	Yes	Unc	89%
**Zhang (2022)[a,b]**	No	SI	0.91 (0.87-0.94)	95.2 (89.1-98.4)	54.5 (46.9-62.0)	Yes	Unc	89%
**Czekierdowski (2023)**	Yes	NR	NR	NR	NR	No	High	54%
**Hu (2023)**	No	CCA	NR	NR	NR	No	High	61%
**Pelayo (2023) [a,b]**	Yes	CCA	0.88	95.1 (88.7-100)	74.1 (65.9-82.3)	No	High	61%
**Pelayo (2023) [a,b]**	No	CCA	0.84	87.8 (78.4-97.2)	67.9 (59.5-76.3)	No	High	61%
**Rashmi (2023) [c]**	Yes	No missings	0.8	NR	NR	No	High	57%
**Rashmi (2023) [c]**	No	No missings	0.786	NR	NR	No	High	57%
**Wang And Yang (2023) [b]**	Yes	CCA	0.925 (0.897-0.948)	94.4 (90.0-97.3)	90.6 (86.4-93.8)	No	High	50%

NR, not reported; Unc, unclear; CCA, complete case analysis; SI, single imputation; MI, multiple imputation; Calibr, calibration; ADNEX, Assessment of Different NEoplasias in the adneXa; AUC, Area under the receiver operating characteristic curve; RoB, Risk of bias; TRIPOD, Transparent reporting of a multivariable prediction model for individual prognosis or diagnosis

[a,b] Included in meta-analysis for AUC (a) and/or sensitivity and specificity (b) in operated patients.

[c] Not included in meta-analysis for AUC because they presented an AUC after dichotomising or categorising risks, i.e. the ROC curve has only 1 or a few points. This was either clear from the ROC curve that was shown, or from the fact that the AUC equals the average of sensitivity and specificity.

Table S5. Reported performance metrics or graphs.

Metric/graph	All 47 studies, n (%)	36 studies without histological or clinical focus, n (%)
Discrimination benign vs malignant (any)	34 (72)	31 (86)
AUC benign vs malignant	34 (72)[a]	31 (86)[a]
Receiver operating characteristic curve shown	31 (66)	27 (75)
Classification benign vs malignant (any)	41 (87)	34 (94)
Sensitivity and specificity for malignancy at the 10% cut-off	31 (66)	28 (50)
Sensitivity and specificity for malignancy at other cut-offs	29 (62)	24 (67)
Positive and negative predictive value at the 10% cut-off	22 (47)	21 (58)
Positive and negative predictive value at any cut-offs	15 (32)	14 (39)
Positive and negative likelihood ratio at any cut-offs	18 (36)	15 (42)
Diagnostics odds ratio (DOR) at the 10% cut-off	10 (21)	10 (28)
Accuracy	8 (17)	6 (17)
Multinomial discrimination (any)	12 (26)	12 (33)
Pairwise AUC	12 (26)[b]	12 (33)[b]
Polytomous Discrimination Index (PDI)	3 (6)	3 (8)
Calibration (any)	4 (9)	4 (11)
Calibration plot for risk of malignancy	4 (9)	4 (11)
Calibration intercept and slope for risk of malignancy	1 (2)	1 (3)
Multinomial calibration plots	1 (2)	1 (3)
Clinical utility (any)	1 (2)	1 (3)
Net benefit	1 (2)	1 (3)
Decision curve (Net benefit over different thresholds)	1 (2)	1 (3)

AUC; Area under the receiver operating characteristic curve.

[a] Four of these reported the AUC after dichotomising or categorising risk (See Table S4 for details).

[b] Four of these reported the methodology for calculating pairwise AUCs.

Barreñada L, et al. BMJMED 2024; 3:e000817. doi: 10.1136/bmjmed-2023-000817

Table S6. Descriptive data for the meta-analysis of area under the receiver operating characteristic curve (AUC) for benign vs malignant tumours.

Meta-analysis	Studies	Centres	Countries	Patients	TRIPOD adherence	References
Main analysis						
Operated patients, with CA125[a]	21	43	18	9202	67%	[15–17,22–26,31,36,43–45,47,49,52,56,58,61,64,65]
Operated patients, without CA125	12	31	13	6309	71%	[15,16,18,25,43–45,49,52,53,56,61]
Sensitivity analyses (operated patients)						
High/unclear RoB studies, with CA125	19	23	14	6103	65%	[17,18,22–26,31,36,43–45,47,49,52,56,58,61,64]
Low RoB studies, with CA125	2	20	7	3099	88%	[15,16]
IOTA studies, with CA125	4	23	8	3752	73%	[15,16,23,26]
Non-IOTA studies, with CA125	17	20	13	5450	66%	[17,22,24,25,31,36,43–45,47,49,52,56,58,61,64,65]
High/unclear RoB studies, without CA125	10	11	7	3210	68%	[18,25,43–45,49,52,53,56,61]
Low RoB studies, without CA125	2	20	7	3099	88%	[15,16]
IOTA studies, without CA125	2	20	7	3099	88%	[15,16]
Non-IOTA studies, without CA125	10	11	7	3210	68%	[18,25,43–45,49,52,53,56,61]
Subgroup analyses (operated patients)						
Asian centres, with CA125	11	13	7	4009	66%	[18,36,43–45,47,52,56,58,58,64]
Asian centres, without CA125	7	8	4	2711	71%	[18,43–45,49,52,56]
Chinese centres, with CA125	5	5	1	1988	73%	[18,66–69]
Chinese centres, without CA125	4	4	1	1612	74%	[18,66–68]
European centres, with CA125	8	28	9	4835	70%	[15–17,22,26,31,61]
European centres, without CA125	3	21	7	3221	79%	[15,16,61]
Non-oncology centres, with CA125	2	9	4	1327	82%	[16,17]
Non-oncology centres, without CA125	1	8	4	750	100%	[16]
Oncology centres, with CA125	18	31	16	7306	68%	[24,47,49,25,52,56,64,43–45,23,36,58,15,16,65,31,17,61]
Oncology centres, without CA125	12	23	13	5559	71%	[16,18,25,43–45,49,52,53,56,61]
Postmenopausal, with CA125	11	32	14	2359	69%	[15,16,22,24–26,31,31,36,44,47]
Postmenopausal, without CA125	4	22	9	1623	79%	[15,16,25,44]
Premenopausal, with CA125	11	32	14	3061	69%	[15,16,22,24–26,31,31,36,44,47]
Premenopausal, without CA125	4	22	9	2060	79%	[15,16,25,44]
Target population						
Operated and non-surgically managed patients, with CA125	2	18	8	5167	80%	[16,51]
Operated and non-surgically managed patients, without CA125[b]	1	17	7	4905	100%	[16]

[a] Szubert (2016) indicated the use of ADNEX version without CA125 in 9 patients where the variable was missing but the results were pooled in the ADNEX with CA125 meta-analysis. [b] Results from [16].

AUC, area under the receiver operating characteristic curve; RoB, risk of bias; TRIPOD, Transparent reporting of a multivariable prediction model for individual prognosis or diagnosis

Table S7. Descriptive data for the meta-analyses of sensitivity and specificity and clinical utility at the 10% risk of malignancy threshold in operated patients.

Meta-analysis	Studies	Centres	Countries	Patients	TRIPOD adherence	References
Main analysis						
Operated patients, with CA125 [a]	23	44	17	9989	67%	[15–18,22–25,27,31,33,36,43–45,49,52,55,56,58,61,63]
Operated patients, without CA125	10	29	13	5465	73%	[15,16,18,25,44,45,49,53,56,61]
Sensitivity analyses						
High/unclear RoB studies, with CA125	21	24	13	6890	64%	[17,18,22–27,31,33,36,43–45,49,52,55,56,58,61,63]
Low RoB studies, with CA125	2	20	7	3099	93%	[15,16]
IOTA studies, with CA125	4	23	8	3752	76%	[15,16,23,26]
Non-IOTA studies, with CA125	19	21	12	6237	65%	[17,18,22,24,25,27,31,33,36,43–45,49,52,55,56,58,61,63]
High/unclear RoB studies, without CA125	8	9	7	2366	68%	[18,25,44,45,49,53,56]
Low RoB studies, without CA125	3	21	8	3381	92%	[15,16,18]
IOTA studies, without CA125	2	20	7	3099	93%	[15,16]
Non-IOTA studies, without CA125	8	9	7	2366	68%	[18,25,44,45,49,53,56,61]
Subgroup analyses						
Asian centres, with CA125	13	14	6	4796	65%	[18,27,33,36,43–45,49,52,55,56,58,63]
Asian centres, without CA125	5	6	4	1867	71%	[18,44,45,49,56]
Chinese centres, with CA125	7	7	1	3167	67%	[18,66–71]
European centres, with CA125	8	28	9	4835	71%	[15–17,22,23,26,61]
European centres, without CA125	3	21	7	3221	82%	[15,16,61]
Non-oncology centres, with CA125	2	9	4	1327	82%	[16,17]
Non-oncology centres, without CA125	1	8	4	750	100%	[16]
Oncology centres, with CA125	18	30	16	6914	69%	[16,24–27,33,43–45,49,52,56,61,63]
Oncology centres, without CA125	10	21	13	4715	73%	[15,16,18,25,44,45,49,53,56,61]
Postmenopausal, with CA125	10	28	14	2240	68%	[16,22,24–26,31,31,36,44,63]
Postmenopausal, without CA125	4	20	10	1431	77%	[16,25,44,53]
Premenopausal, with CA125	10	28	14	2731	68%	[16,22–26,31,36,44,63]
Premenopausal, without CA125	4	20	10	1792	76%	[16,25,44,53]

[a] Szubert (2016) indicated the use of ADNEX version without CA125 in 9 patients where the variable was missing, but we pooled the results in the ADNEX with CA125 meta-analysis

RoB, risk of bias; TRIPOD, Transparent reporting of a multivariable prediction model for individual prognosis or diagnosis

Table S8. Meta-analysis results of sensitivity and specificity at the 10% risk of malignancy threshold in operated patients.

Meta-analysis	Sensitivity			Specificity		
	Summary estimate (95% CI)[a]	95% PI[b]	τ^2	Summary estimate (95% CI)[a]	95% PI[b]	τ^2
Main analysis						
Operated patients, with CA125	0.94 (0.92-0.95)	0.80 - 0.98	0.37	0.77 (0.73-0.81)	0.47 - 0.93	0.41
Operated patients, without CA125	0.93 (0.90-0.95)	0.73 - 0.99	0.58	0.75 (0.70-0.79)	0.46 - 0.91	0.35
Sensitivity analysis						
High/unclear RoB studies, with CA125	0.93 (0.92-0.95)	0.88 - 0.96	0.07	0.78 (0.72-0.82)	0.46 - 0.94	0.45
Low RoB studies, with CA125	0.94 (0.89-0.96)	0.63 - 0.99	0.94	0.75 (0.68-0.81)	0.43 - 0.92	0.37
IOTA studies, with CA125	0.94 (0.90-0.96)	0.69 - 0.99	0.82	0.74 (0.68-0.79)	0.46 - 0.90	0.30
Non-IOTA studies, with CA125	0.93 (0.91-0.94)	0.88 - 0.96	0.07	0.79 (0.73-0.84)	0.45 - 0.94	0.48
High/unclear RoB studies, without CA125	0.93 (0.90-0.95)	0.89 - 0.96	<0.01	0.76 (0.67-0.84)	0.37 - 0.94	0.42
Low RoB studies, without CA125	0.94 (0.90-0.96)	0.65 - 0.99	0.88	0.73 (0.66-0.79)	0.42 - 0.91	0.35
IOTA studies, without CA125	0.94 (0.89-0.96)	0.61 - 0.99	0.96	0.74 (0.67-0.80)	0.44 - 0.91	0.33
Non-IOTA studies, without CA125	0.93 (0.90-0.95)	0.89 - 0.96	<0.01	0.76 (0.67-0.84)	0.37 - 0.94	0.42
Subgroup analyses						
Asian centres, with CA125	0.93 (0.91-0.95)	0.88 - 0.96	0.07	0.82 (0.75-0.87)	0.48 - 0.96	0.47
Asian centres, without CA125	0.94 (0.90-0.96)	0.88 - 0.97	<0.01	0.79 (0.65-0.88)	0.21 - 0.98	0.58
Chinese centres, with CA125	0.93 (0.91-0.95)	0.85 - 0.97	0.09	0.81 (0.70-0.89)	0.31 - 0.98	0.67
European centres, with CA125	0.95 (0.91-0.97)	0.67 - 0.99	0.97	0.74 (0.68-0.78)	0.47 - 0.90	0.29
European centres, without CA125	0.93 (0.89-0.96)	0.62 - 0.99	0.90	0.73 (0.67-0.79)	0.45 - 0.90	0.30
Non-oncology centres, with CA125	0.88 (0.83-0.91)	0.81 - 0.92	<0.01	0.83 (0.77-0.87)	0.64 - 0.93	0.10
Non-oncology centres, without CA125	0.87 (0.77-0.93)	0.52 - 0.97	0.20	0.83 (0.78-0.87)	0.67 - 0.92	0.05
Oncology centres, with CA125	0.95 (0.93-0.96)	0.81 - 0.99	0.48	0.73 (0.68-0.78)	0.41 - 0.91	0.41
Oncology centres, without CA125	0.94 (0.92-0.96)	0.77 - 0.99	0.53	0.72 (0.66-0.77)	0.42 - 0.90	0.33
Postmenopausal, with CA125	0.97 (0.94-0.98)	0.69 - >.99	1.48	0.65 (0.58-0.71)	0.36 - 0.86	0.31
Postmenopausal, without CA125	0.96 (0.92-0.98)	0.59 - >.99	1.69	0.62 (0.54-0.68)	0.36 - 0.82	0.21
Premenopausal, with CA125	0.93 (0.87-0.96)	0.41 - >.99	1.90	0.81 (0.75-0.86)	0.47 - 0.95	0.56
Premenopausal, without CA125	0.90 (0.84-0.94)	0.64 - 0.98	0.50	0.82 (0.76-0.86)	0.55 - 0.94	0.34

CI, confidence interval; PI, prediction interval
[a] CI estimated using Wilson's interval **[6]** when not reported
[b] PI calculated as in [72].

Table S9. Meta-analysis results of clinical utility at the 10% cut-off in operated patients.

Meta-analysis	Net Benefit: Summary estimate (95% CrI)[a]	Net Benefit: 95% PI[b]	Relative utility: Summary estimate (95% CrI)[a]	Relative utility: 95% PI[b]	P(useful)[b]
Main analysis					
Operated patients, with CA125	0.28 (0.22-0.33)	0.05 - 0.65	0.54 (0.45-0.61)	-0.12 - 0.78	95%
Operated patients, without CA125	0.28 (0.21-0.35)	0.05 - 0.68	0.50 (0.37-0.62)	-0.44 - 0.79	91%
Sensitivity analyses					
High/unclear RoB studies, with CA125	0.25 (0.19-0.31)	0.06 - 0.58	0.57 (0.46-0.67)	-0.19 - 0.83	95%
Low RoB studies, with CA125	0.32 (0.22-0.43)	0.04 - 0.75	0.45 (0.25-0.61)	-0.66 - 0.78	89%
IOTA studies, with CA125	0.32 (0.23-0.41)	0.06 - 0.70	0.48 (0.33-0.60)	-0.60 - 0.77	91%
Non-IOTA studies, with CA125	0.24 (0.17-0.31)	0.05 - 0.60	0.57 (0.44-0.67)	-0.35 - 0.83	93%
High/unclear RoB studies, without CA125	0.21 (0.15-0.28)	0.07 - 0.45	0.57 (0.33-0.73)	-0.71 - 0.86	90%
Low RoB studies, without CA125	0.32 (0.23-0.43)	0.04 - 0.72	0.43 (0.24-0.57)	-0.76 - 0.77	87%
IOTA studies, without CA125	0.32 (0.23-0.42)	0.04 - 0.74	0.44 (0.24-0.59)	-1.00 - 0.77	85%
Non-IOTA studies, without CA125	0.21 (0.15-0.30)	0.07 - 0.48	0.57 (0.34-0.72)	-0.47 - 0.85	91%
Subgroup analyses					
Asian centres, with CA125	0.22 (0.15-0.30)	0.04 - 0.57	0.62 (0.47-0.73)	-0.49 - 0.86	92%
Asian centres, without CA125	0.19 (0.11-0.30)	0.04 - 0.50	0.62 (0.31-0.81)	-1.08 - 0.90	88%
Chinese centres, with CA125	0.30 (0.2-0.41)	0.08 - 0.60	0.50 (0.15-0.71)	-1.10 - 0.86	85%
European centres, with CA125	0.30 (0.22-0.38)	0.05 - 0.68	0.49 (0.37-0.58)	-0.23 - 0.73	96%
European centres, without CA125	0.32 (0.23-0.43)	0.05 - 0.73	0.41 (0.23-0.56)	-0.74 - 0.76	86%
Non-oncology centres, with CA125	0.18 (0.1-0.29)	0.01 - 0.48	0.51 (0.07-0.75)	-1.78 - 0.83	86%
Non-oncology centres, without CA125	0.18 (0.08-0.32)	0.01 - 0.55	0.50 (-0.04-0.78)	-1.98 - 0.85	85%
Oncology centres, with CA125	0.32 (0.26-0.38)	0.10 - 0.65	0.50 (0.39-0.59)	-0.27 - 0.78	94%
Oncology centres, without CA125	0.32 (0.24-0.41)	0.06 - 0.70	0.47 (0.31-0.60)	-0.57 - 0.80	88%
Postmenopausal, with CA125	0.42 (0.32-0.52)	0.07 - 0.82	0.39 (0.20-0.54)	-1.28 - 0.77	83%
Postmenopausal, without CA125	0.44 (0.35-0.54)	0.11 - 0.78	0.34 (0.09-0.53)	-1.21 - 0.73	79%
Premenopausal, with CA125	0.2 (0.14-0.26)	0.03 - 0.59	0.58 (0.45-0.69)	-0.27 - 0.84	94%
Premenopausal, without CA125	0.19 (0.11-0.29)	0.01 - 0.70	0.57 (0.35-0.72)	-1.47 - 0.83	87%

CrI, credible interval; PI, prediction interval

[a] CrI estimated using Bayesian sampling methods

[b] PI calculated using trivariate meta-analysis as in Wynants et al [13]

[c] P(useful) is the probability that the Net Benefit of using the model in a new random centre at the 10% threshold is superior to those of the baseline strategies of treating all or treating none of the patients.

Table S10. Descriptive data for the meta-analysis of pairwise area under the receiver operating characteristic curve (AUC) calculated with conditional risk method in operated patients.

Meta-analysis	Studies	Centres	Countries	N first group	N second group	TRIPOD adherence	References
ADNEX with CA125							
Benign vs Borderline	5	23	9	2879	295	83%	**[15–17,44,65]**
Benign vs Stage I	5	23	9	2879	306	83%	**[15–17,44,65]**
Benign vs Stage II-IV	5	23	9	2879	644	83%	**[15–17,44,65]**
Benign vs Metastatic	4	22	8	2583	186	85%	**[15–17,65]**
Borderline vs Stage I	5	23	9	295	306	83%	**[15–17,44,65]**
Borderline vs Stage II-IV	5	23	9	295	644	83%	**[15–17,44,65]**
Borderline vs Metastatic	4	22	8	284	186	85%	**[15–17,65]**
Stage I vs Stage II-IV	5	23	9	306	644	83%	**[15–17,44,65]**
Stage I vs Metastatic	4	22	8	286	186	85%	**[15–17,65]**
Stage II-IV vs Metastatic	4	22	8	619	186	85%	**[15–17,65]**
ADNEX without CA125							
Benign vs Borderline	4	22	9	2446	269	88%	**[15,16,44,65]**
Benign vs Stage I	4	22	9	2446	267	88%	**[15,16,44,65]**
Benign vs Stage II-IV	4	22	9	2446	586	88%	**[15,16,44,65]**
Benign vs Metastatic	3	21	8	2150	165	92%	**[15,16,65]**
Borderline vs Stage I	4	22	9	269	267	88%	**[15,16,44,65]**
Borderline vs Stage II-IV	4	22	9	269	586	88%	**[15,16,44,65]**
Borderline vs Metastatic	3	21	8	258	165	92%	**[15,16,65]**
Stage I vs Stage II-IV	4	22	9	267	586	88%	**[15,16,44,65]**
Stage I vs Metastatic	3	21	8	247	165	92%	**[15,16,65]**
Stage II-IV vs Metastatic	3	21	8	561	165	92%	**[15,16,65]**

Pairwise analysis including metastatic tumours includes 1 study less because **[44]** had too few metastases.
TRIPOD, Transparent reporting of a multivariable prediction model for individual prognosis or diagnosis

Table S11. Meta-analysis results of pairwise area under receiver operating characteristic curve (AUC) with conditional risk method in operated patients.

Meta-Analysis	Studies	Summary estimate	95% CI	95% PI	τ^2
ADNEX with CA125					
Benign vs Borderline	5	0.86	0.83 to 0.90	0.80 to 0.93	0.06
Benign vs Stage I	5	0.92	0.88 to 0.96	0.82 to >.99	0.34
Benign vs Stage II-IV	5	0.98	0.97 to 0.99	0.96 to >.99	0.18
Benign vs Metastatic	4	0.95	0.92 to 0.98	0.90 to >.99	0.22
Borderline vs Stage I	5	0.72	0.61 to 0.82	0.49 to 0.92	0.24
Borderline vs Stage II-IV	5	0.90	0.86 to 0.94	0.82 to 0.97	0.12
Borderline vs Metastatic	4	0.87	0.80 to 0.93	0.74 to 0.97	0.18
Stage I vs Stage II-IV	5	0.82	0.75 to 0.88	0.68 to 0.94	0.16
Stage I vs Metastatic	4	0.78	0.70 to 0.85	0.64 to 0.90	0.10
Stage II-IV vs Metastatic	4	0.78	0.71 to 0.85	0.65 to 0.90	0.09
ADNEX without CA125					
Benign vs Borderline	4	0.86	0.81 to 0.91	0.77 to 0.95	0.10
Benign vs Stage I	4	0.92	0.88 to 0.97	0.82 to >.99	0.33
Benign vs Stage II-IV	4	0.97	0.95 to 0.98	0.94 to 0.99	0.17
Benign vs Metastatic	3	0.94	0.91 to 0.99	0.87 to >.99	0.46
Borderline vs Stage I	4	0.73	0.60 to 0.85	0.48 to 0.94	0.29
Borderline vs Stage II-IV	4	0.89	0.84 to 0.93	0.79 to 0.96	0.12
Borderline vs Metastatic	3	0.89	0.81 to 0.96	0.77 to >.99	0.30
Stage I vs Stage II-IV	4	0.75	0.63 to 0.85	0.51 to 0.95	0.30
Stage I vs Metastatic	3	0.78	0.68 to 0.90	0.61 to 0.96	0.22
Stage II-IV vs Metastatic	3	0.66	0.50 to 0.81	0.39 to 0.90	0.27

Pairwise analysis including metastatic tumours includes 1 study less because one study **[44]** had too few metastases.
CI, confidence interval; PI, prediction interval

SUPPLEMENTARY FIGURES

Figure S1. TRIPOD (Transparent Reporting of a multivariable prediction model for Individual Prognosis Or Diagnosis) adherence per study (N = 47).

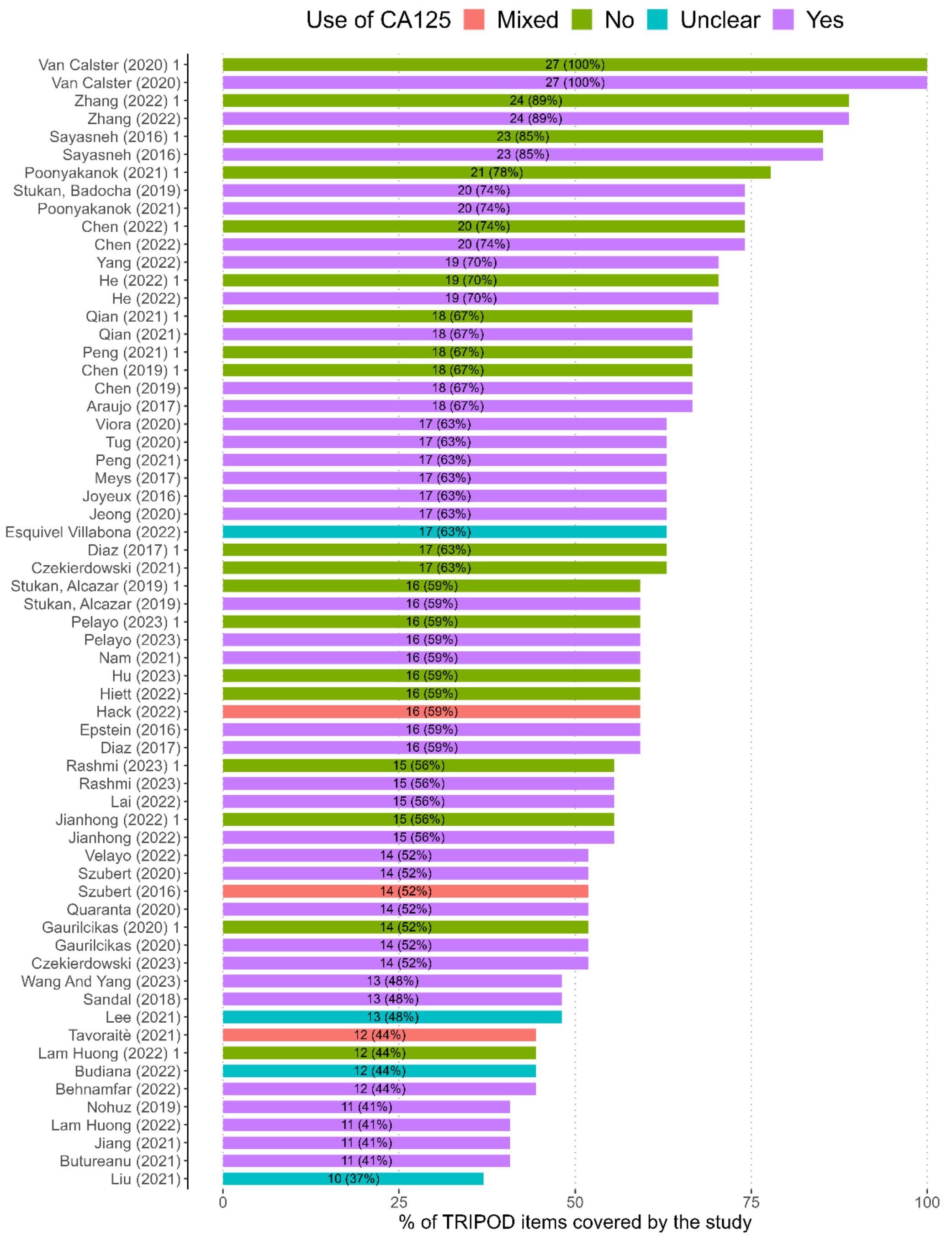

25

Figure S2. PROBAST (Prediction model study Risk Of Bias ASsessment Tool) results by subdomain and overall for studies evaluating ADNEX without CA125. Figure generated adapting code from [73]. Blue rows refer to studies included in meta-analysis for the AUC and green rows refer to studies that are included in all meta-analysis.

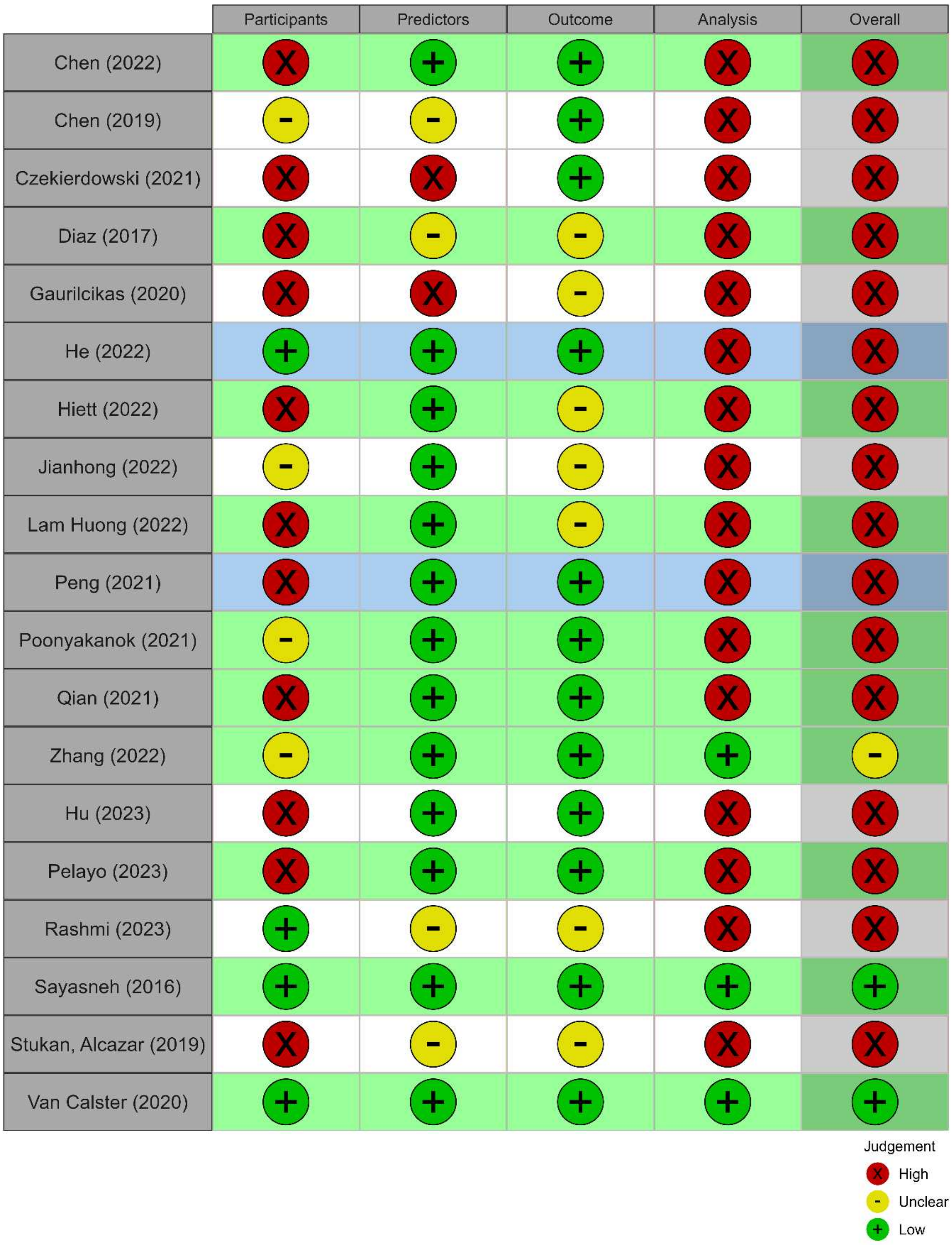

Figure S3. PROBAST (Prediction model study Risk Of Bias ASsessment Tool) results by subdomain and overall for studies evaluating ADNEX with CA125. Figure generated adapting code from [73]. Yellow rows refer to studies that are included in meta-analysis for specificity, sensitivity and clinical utility, blue rows refer to studies included in meta-analysis for the AUC and green rows refer to studies that are included in all meta-analysis.

	Participants	Predictors	Outcome	Analysis	Overall
Araujo (2017)	High	Unclear	Low	High	High
Behnamfar (2022)	High	Low	Low	High	High
Butureanu (2021)	High	Unclear	Unclear	High	High
Chen (2022)	High	Unclear	Low	High	High
Chen (2019)	Unclear	Unclear	Low	High	High
Czekierdowski (2023)	High	Unclear	High	High	High
Diaz (2017)	High	Unclear	Unclear	High	High
Gaurilcikas (2020)	High	High	Unclear	High	High
Hack (2022)	High	Unclear	Unclear	High	High
He (2022)	Low	Low	Low	High	High
Jeong (2020)	High	Low	High	High	High
Jiang (2021)	High	Unclear	Unclear	High	High
Jianhong (2022)	Unclear	Low	Unclear	High	High
Joyeux (2016)	High	Unclear	Low	High	High
Lai (2022)	High	Unclear	Unclear	High	High
Lam Huong (2022)	High	Unclear	Unclear	High	High
Nam (2021)	High	Unclear	Unclear	High	High
Nohuz (2019)	High	Unclear	Unclear	High	High
Peng (2021)	High	Unclear	Low	High	High
Poonyakanok (2021)	Unclear	Unclear	Low	High	High
Qian (2021)	High	Low	Low	High	High
Quaranta (2020)	High	Unclear	Unclear	High	High
Sandal (2018)	High	Unclear	Unclear	High	High
Stukan, Badocha (2019)	Unclear	Low	Low	High	High
Szubert (2020)	High	Unclear	Unclear	High	High
Tavoraitè (2021)	High	Unclear	Unclear	High	High
Tug (2020)	Unclear	Unclear	Unclear	High	High
Velayo (2022)	High	Low	Unclear	High	High
Viora (2020)	High	Unclear	Unclear	High	High
Yang (2022)	Unclear	Unclear	Low	High	High
Zhang (2022)	Unclear	Low	Low	Low	Unclear
Pelayo (2023)	High	Low	Low	High	High
Rashmi (2023)	Low	Unclear	Unclear	High	High
Wang And Yang (2023)	High	Unclear	Unclear	High	High
Szubert (2016)	High	Unclear	Unclear	High	High
Epstein (2016)	Unclear	Unclear	Unclear	High	High
Sayasneh (2016)	Low	Low	Low	Low	Low
Stukan, Alcazar (2019)	High	Unclear	Unclear	High	High
Meys (2017)	High	Unclear	Unclear	High	High
Van Calster (2020)	Low	Low	Low	Low	Low

Judgement
X High
- Unclear
+ Low

Barreñada L, *et al. BMJMED* 2024; 3:e000817. doi: 10.1136/bmjmed-2023-000817

Figure S4. PROBAST (Prediction model study Risk Of Bias ASsessment Tool) results by subdomain and overall for studies evaluating ADNEX but for which it was unclear whether the version with or without CA125 was used. Figure generated adapting code from [73].

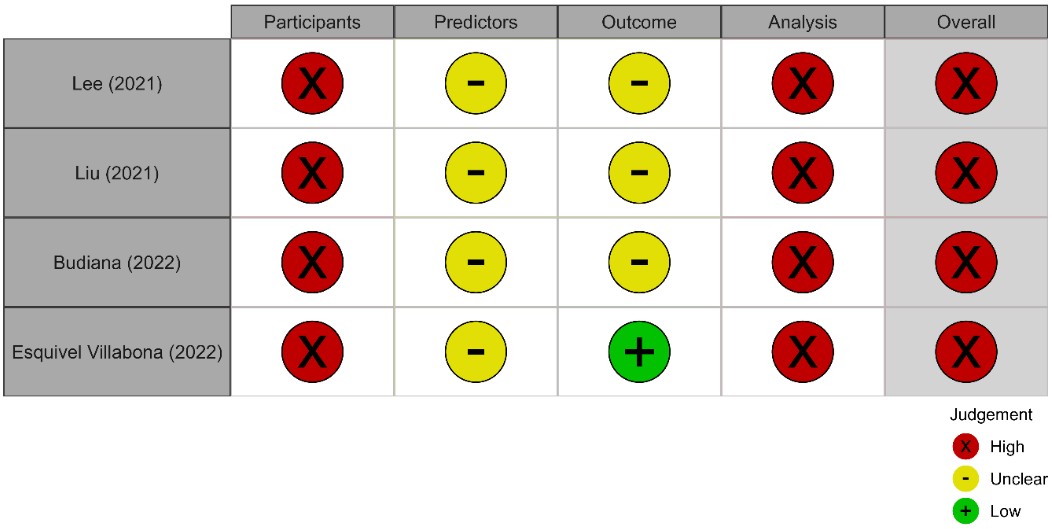

Figure S5. PROBAST (Distribution of Prediction model study Risk Of Bias ASsessment Tool) results per signalling question in all 63 validations. For PROBAST tool signalling questions see [74,75]. Percentages may sum to 99 or 101 due to rounding.

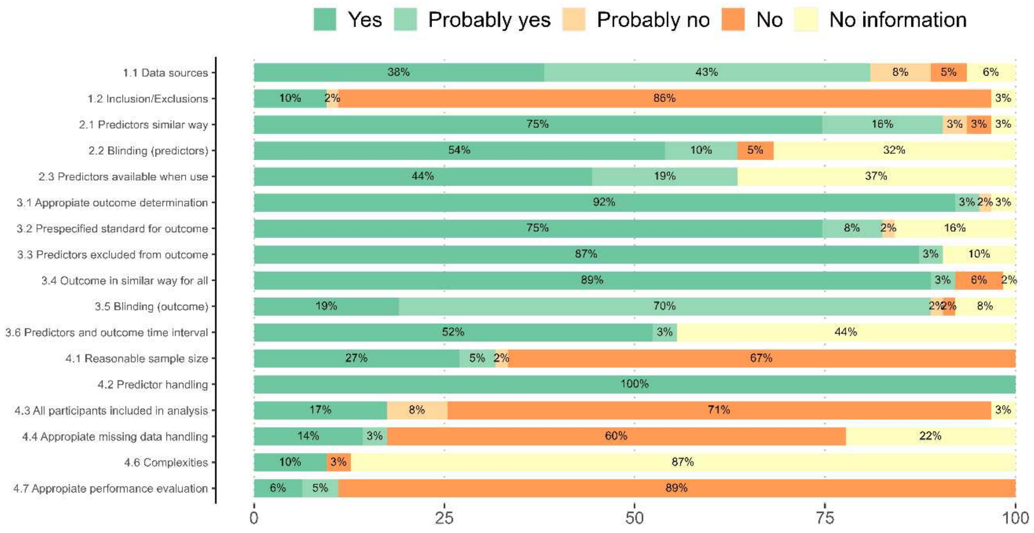

Barreñada L, *et al. BMJMED* 2024; 3:e000817. doi: 10.1136/bmjmed-2023-000817

Figure S6. Forest plot of Net Benefit for ADNEX without CA125. CrI, credible interval; NB, net benefit; Prev, prevalence of malignancy

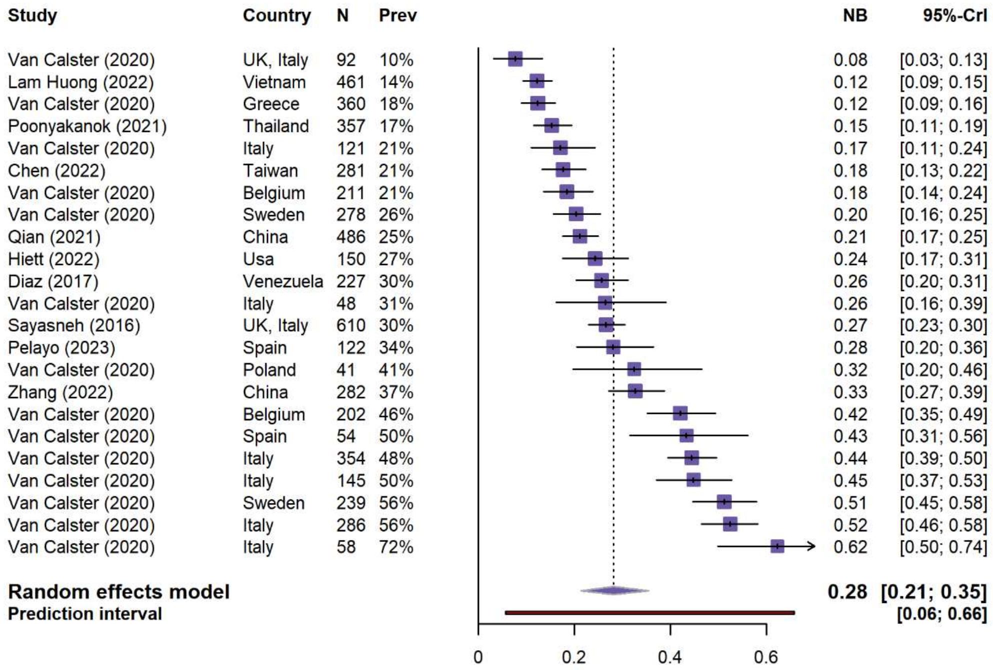

Barreñada L, *et al. BMJMED* 2024; 3:e000817. doi: 10.1136/bmjmed-2023-000817

Figure S7. Forest plot of Relative Utility for ADNEX with CA125. CrI, credible interval; Prev, prevalence of malignancy; RU, relative utility

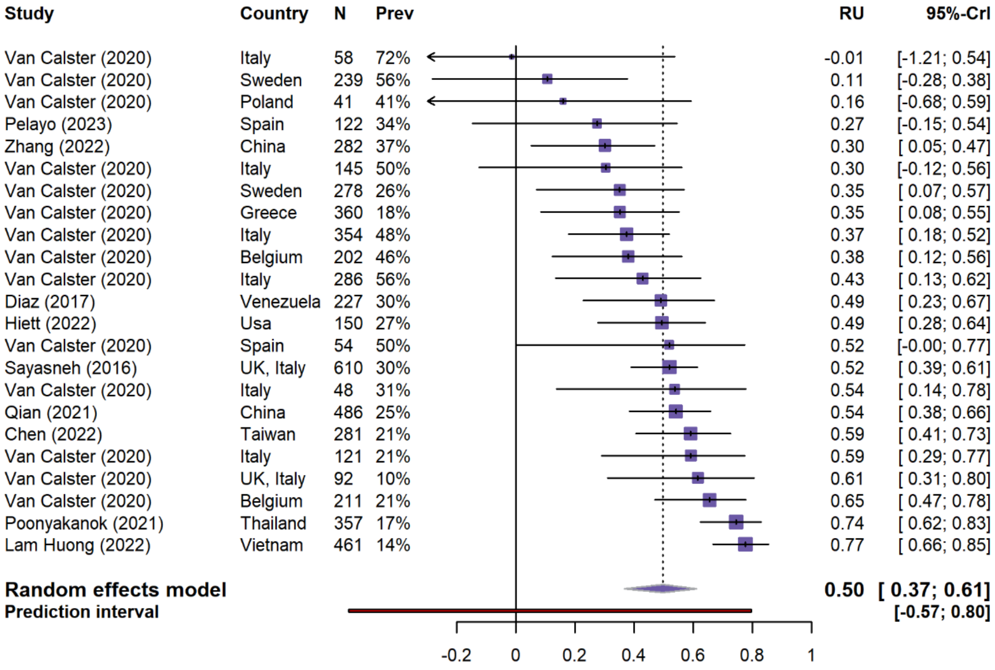

Figure S8. Forest plot of Net Benefit for ADNEX with CA125. CrI, credible interval; NB, net benefit; Prev, prevalence of malignancy

Study	Country	N	Prev	NB	95%-CrI
Tug (2020)	Turkey	285	9%	0.07	[0.04; 0.11]
Van Calster (2020)	UK, Italy	92	10%	0.08	[0.04; 0.14]
Joyeux (2016)	France	284	11%	0.09	[0.05; 0.12]
Lam Huong (2022)	Vietnam	461	14%	0.12	[0.09; 0.15]
Van Calster (2020)	Greece	360	18%	0.13	[0.09; 0.17]
Poonyakanok (2021)	Thailand	357	17%	0.16	[0.12; 0.19]
Jeong (2020)	South Korea	54	19%	0.18	[0.09; 0.28]
Chen (2022)	Taiwan	281	21%	0.18	[0.13; 0.22]
Van Calster (2020)	Italy	121	21%	0.18	[0.11; 0.25]
Van Calster (2020)	Belgium	211	21%	0.18	[0.13; 0.23]
Viora (2020)	Italy	577	25%	0.21	[0.17; 0.24]
Lai (2022)	China	734	23%	0.21	[0.18; 0.24]
Qian (2021)	China	486	25%	0.21	[0.18; 0.25]
Van Calster (2020)	Sweden	278	26%	0.21	[0.16; 0.26]
Sandal (2018)	Turkey	191	28%	0.25	[0.18; 0.31]
Szubert (2016)	Spain	123	28%	0.25	[0.17; 0.33]
Diaz (2017)	Venezuela	227	30%	0.26	[0.21; 0.32]
Sayasneh (2016)	UK, Italy	610	30%	0.27	[0.23; 0.30]
Van Calster (2020)	Italy	48	31%	0.27	[0.17; 0.39]
Yang (2022)	China	376	31%	0.27	[0.23; 0.32]
Pelayo (2023)	Spain	122	34%	0.30	[0.22; 0.38]
He (2022)	China	620	35%	0.30	[0.26; 0.34]
Szubert (2016)	Poland	204	34%	0.30	[0.24; 0.37]
Meys (2017)	Netherlands	326	35%	0.32	[0.27; 0.37]
Zhang (2022)	China	282	37%	0.33	[0.27; 0.38]
Van Calster (2020)	Poland	41	41%	0.33	[0.21; 0.46]
Wang And Yang (2023)	China	445	40%	0.37	[0.32; 0.41]
Peng (2021)	China	224	47%	0.42	[0.36; 0.49]
Van Calster (2020)	Belgium	202	46%	0.42	[0.35; 0.49]
Van Calster (2020)	Spain	54	50%	0.43	[0.31; 0.55]
Van Calster (2020)	Italy	354	48%	0.44	[0.39; 0.50]
Van Calster (2020)	Italy	145	50%	0.45	[0.37; 0.52]
Araujo (2017)	Brazil	131	52%	0.46	[0.37; 0.55]
Stukan, Badocha (2019)	Poland	100	50%	0.46	[0.37; 0.56]
Van Calster (2020)	Sweden	239	56%	0.51	[0.45; 0.57]
Van Calster (2020)	Italy	286	56%	0.53	[0.47; 0.59]
Van Calster (2020)	Italy	58	72%	0.63	[0.51; 0.73]
**Random effects model**				**0.27**	**[0.22; 0.33]**
**Prediction interval**					**[0.06; 0.64]**

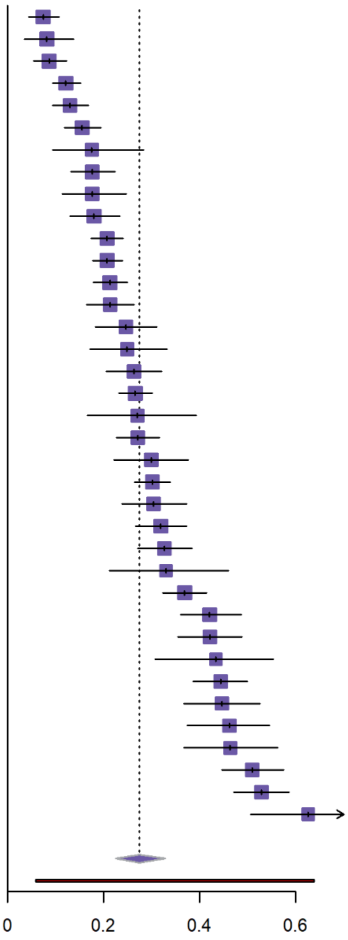

Figure S9. Forest plot of Relative Utility for ADNEX with CA125. CrI, credible interval; Prev, prevalence of malignancy; RU, relative utility

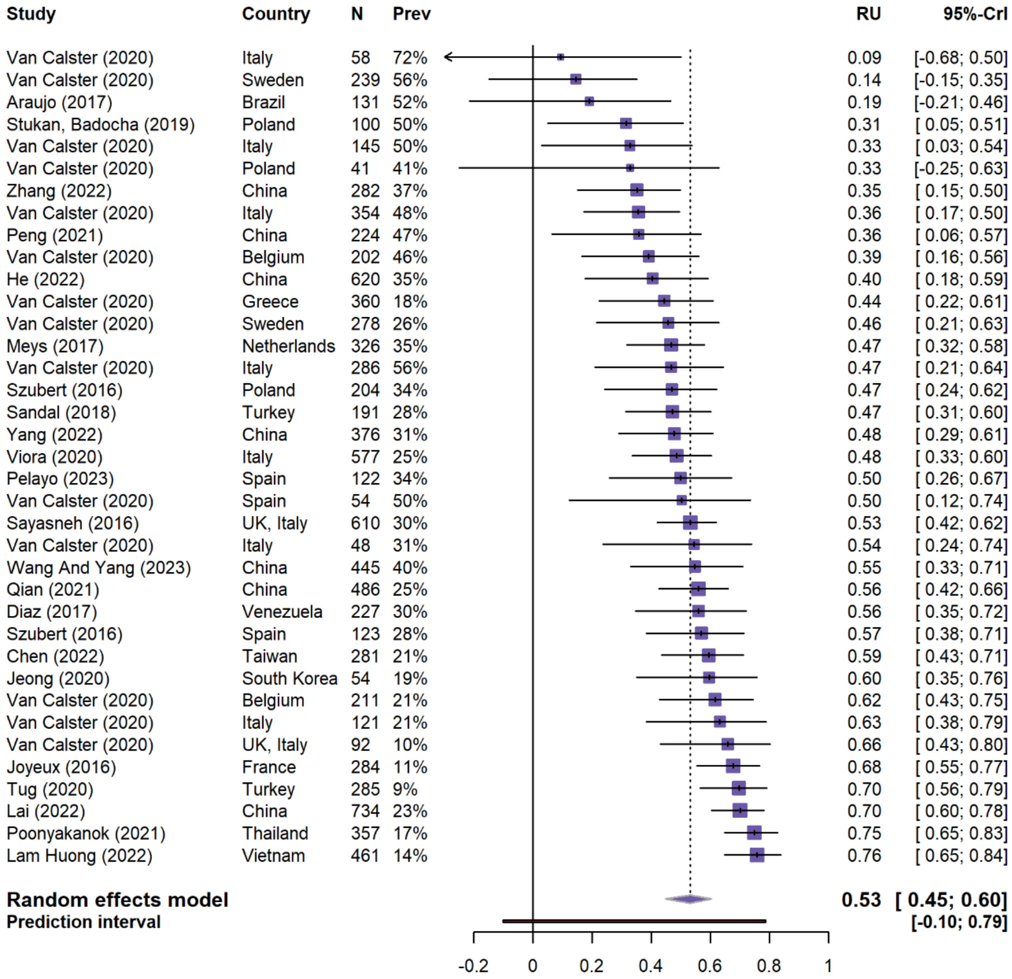

Figure S10. Meta-regression of the area under the receiver operating characteristic curve (AUC) on the centre-specific prevalence of malignancy for ADNEX with CA 125 (left) and without CA125 (rigt).

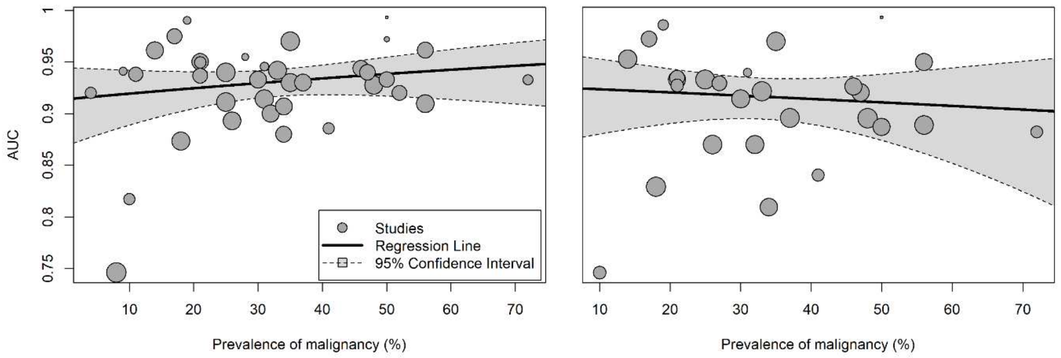

Figure S11. Meta-regression of sensitivity (left) and specificity (right) on the centre-specific prevalence of malignancy for ADNEX with CA125.

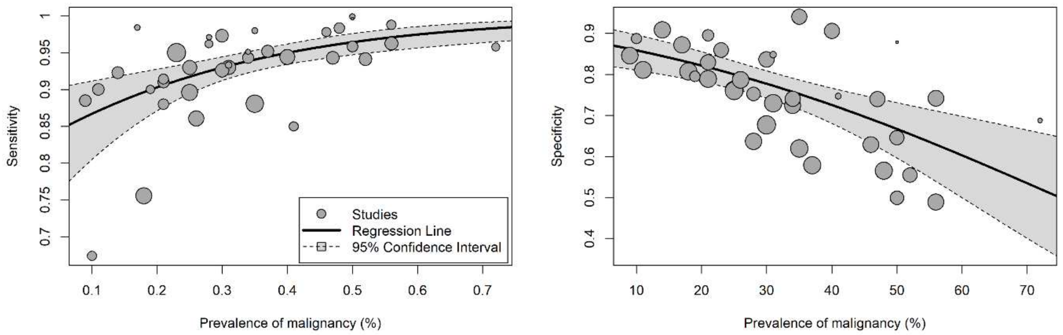

35

Figure S12. Meta-regression of sensitivity (left) and specificity (right) on the centre-specific prevalence of malignancy for ADNEX without CA125.

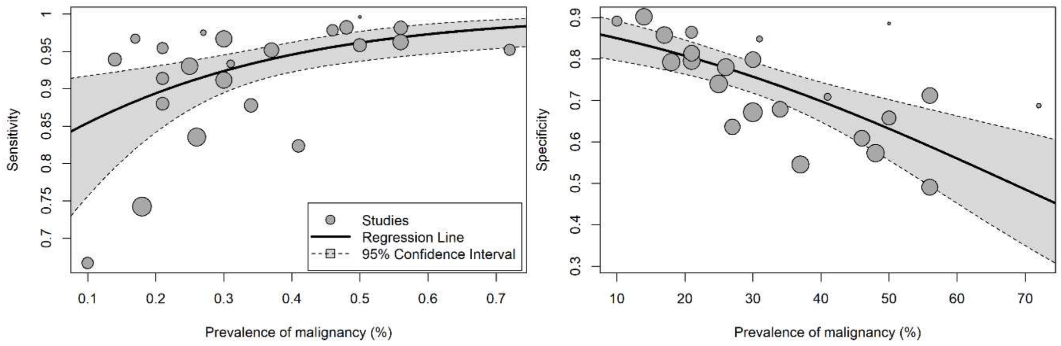

Figure S13. Funnel plots of the AUC for ADNEX with CA125 validation stratified by prevalence of malignancy (left) and PROBAST Risk of Bias (right).

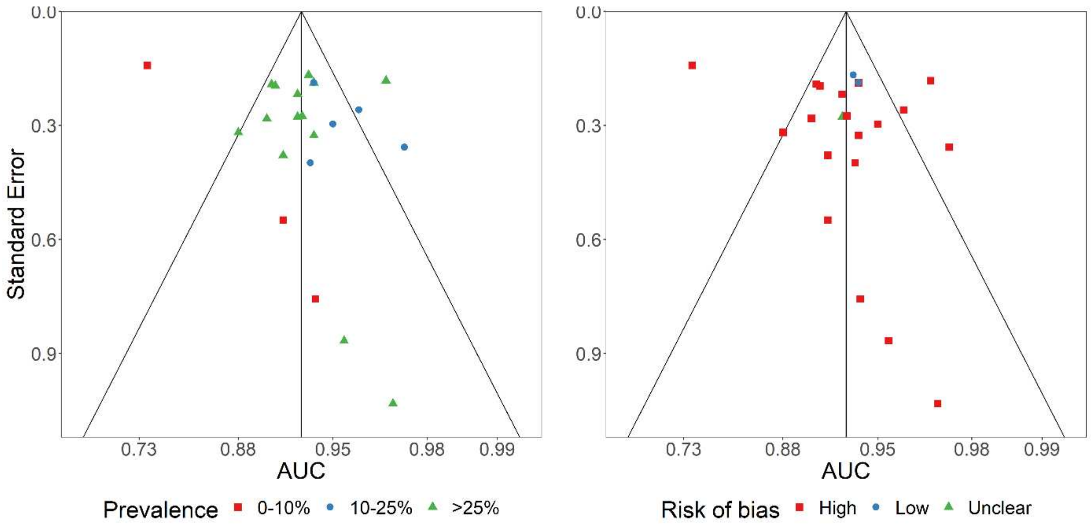

References

1 Van Calster B, Van Hoorde K, Valentin L, *et al.* Evaluating the risk of ovarian cancer before surgery using the ADNEX model to differentiate between benign, borderline, early and advanced stage invasive, and secondary metastatic tumours: prospective multicentre diagnostic study. *BMJ*. 2014;349:g5920.

2 Dirk Timmerman, Lil Valentin, Tom Bourne, *et al.* Terms, definitions and measurements to describe the sonographic features of adnexal tumors: a consensus opinion from the International Ovarian Tumor Analysis (IOTA) group. *Ultrasound Obstet Gynecol*. Published Online First: 2000. doi: 10.1046/j.1469-0705.2000.00287.x

3 Wohlin C. Guidelines for snowballing in systematic literature studies and a replication in software engineering. *Proceedings of the 18th International Conference on Evaluation and Assessment in Software Engineering - EASE '14*. London, England, United Kingdom: ACM Press 2014:1–10. https://doi.org/10.1145/2601248.2601268

4 Newcombe RG. Confidence intervals for an effect size measure based on the Mann–Whitney statistic. Part 2: asymptotic methods and evaluation. *Stat Med*. 2006;25:559–73.

5 Debray TP, Damen JA, Riley RD, *et al.* A framework for meta-analysis of prediction model studies with binary and time-to-event outcomes. *Stat Methods Med Res*. 2019;28:2768–86.

6 Agresti A, Coull BA. Approximate Is Better than 'Exact' for Interval Estimation of Binomial Proportions. *Am Stat*. 1998;52:119–26.

7 Brown LD, Cai TT, DasGupta A. Interval Estimation for a Binomial Proportion. *Stat Sci*. 2001;16:101–33.

8 Doebler P, Holling H. Meta-Analysis of Diagnostic Accuracy with mada. 2017. https://api.semanticscholar.org/CorpusID:30459830

9 Viechtbauer W. Bias and Efficiency of Meta-Analytic Variance Estimators in the Random-Effects Model. *J Educ Behav Stat*. 2005;30:261–93.

10 Harrer M, Cuijpers P, Furukawa TA, *et al. Doing Meta-Analysis With R: A Hands-On Guide*. 1st ed. Boca Raton, FL and London: Chapman & Hall/CRC Press 2021. https://www.routledge.com/Doing-Meta-Analysis-with-R-A-Hands-On-Guide/Harrer-Cuijpers-Furukawa-Ebert/p/book/9780367610074

11 IntHout J, Ioannidis JP, Borm GF. The Hartung-Knapp-Sidik-Jonkman method for random effects meta-analysis is straightforward and considerably outperforms the standard DerSimonian-Laird method. *BMC Med Res Methodol*. 2014;14:25.

12 Cornell JE, Mulrow CD, Localio R, *et al.* Random-Effects Meta-analysis of Inconsistent Effects: A Time for Change. *Ann Intern Med*. 2014;160:267–70.

13 Wynants L, Riley RD, Timmerman D, *et al.* Random-effects meta-analysis of the clinical utility of tests and prediction models. *Stat Med*. 2018;37:2034–52.

14 Bujkiewicz S. Bayesian meta-analytical methods to incorporate multiple surrogate endpoints in drug development process. *Stat Med*. 2022;41:5877–8.

15 Sayasneh A, Ferrara L, De Cock B, *et al.* Evaluating the risk of ovarian cancer before surgery using the ADNEX model: a multicentre external validation study. *Br J Cancer*. 2016;115:542–8.

16 Van Calster B, Valentin L, Froyman W, *et al.* Validation of models to diagnose ovarian cancer in patients managed surgically or conservatively: multicentre cohort study. *BMJ*. 2020;370:m2614.

17 Viora E, Piovano E, Baima Poma C, *et al.* The ADNEX model to triage adnexal masses: An external validation study and comparison with the IOTA two-step strategy and subjective assessment by an experienced ultrasound operator. *Eur J Obstet Gynecol Reprod Biol*. Published Online First: 2020. doi: 10.1016/j.ejogrb.2020.02.022

18 Zhang Y, Zhao Y, Feng L. External Validation of the Assessment of Different NEoplasias in the adneXa Model Performance in Evaluating the Risk of Ovarian Carcinoma Before Surgery in China: A Tertiary Center Study. *J Ultrasound Med*. Published Online First: 2022. doi: 10.1002/jum.15920

19 Moons KGM, Altman DG, Reitsma JB, *et al.* Transparent Reporting of a multivariable prediction model for Individual Prognosis Or Diagnosis (TRIPOD): Explanation and Elaboration. *Ann Intern Med*. 2015;162:W1–73.

20 Gary S Collins, Johannes B Reitsma, Douglas G Altman, *et al.* Transparent reporting of a multivariable prediction model for individual prognosis or diagnosis tripod the tripod statement. *BMJ*. Published Online First: 2015. doi: 10.1136/bmj.g7594

21 Epstein E, Van Calster B, Timmerman D, *et al.* Subjective ultrasound assessment, the ADNEX model and ultrasound-guided tru-cut biopsy to differentiate disseminated primary ovarian cancer from metastatic non-ovarian cancer. *Ultrasound Obstet Gynecol*. Published Online First: 2016. doi: 10.1002/uog.14892

22 Joyeux E, Miras T, Masquin I, *et al.* Before surgery predictability of malignant ovarian tumors based on ADNEX model and its use in clinical practice. *Gynecol Obstet Fertil*. 2016;44:557–64.

23 Szubert S, Wojtowicz A, Moszynski R, *et al.* External validation of the IOTA ADNEX model performed by two independent gynecologic centers. *Gynecol Oncol*. Published Online First: 2016. doi: 10.1016/j.ygyno.2016.06.020

24 Araujo KG, Jales RM, Pereira PN, *et al.* Performance of the IOTA ADNEX model in preoperative discrimination of adnexal masses in a gynecological oncology center. *Ultrasound Obstet Gynecol*. Published Online First: 2017. doi: 10.1002/uog.15963

25 Díaz L, Santos M, Zambrano B, *et al.* Ovarian tumors: Risk of malignancy and IOTA ADNEX model indexes. No technology Doppler diagnostic options [Tumores de ovario: Índices de riesgo de malignidad y modelo ADNEX de IOTA. Opciones diagnósticas sin tecnología doppler]. *Rev Obstet Ginecol Venez*. Published Online First: 2017.

26 Meys EMJ, Jeelof LS, Achten NMJ, *et al.* Estimating risk of malignancy in adnexal masses: external validation of the ADNEX model and comparison with other frequently used ultrasound methods. *Ultrasound Obstet Gynecol Off J Int Soc Ultrasound Obstet Gynecol*. 2017;49:784–92.

27 Sandal K, Polat M, Yassa M, *et al.* Comparision of 'risk of malignancy indices' and 'assesment of different neoplasia in the adnexa' (ADNEX) model as preoperative malignancy evaluation methods for adnexal masses. *Zeynep Kamil Tip Bul*. 2018;49:324–9.

28 Chen H, Qian L, Jiang M, *et al.* Performance of IOTA ADNEX model in evaluating adnexal masses in a gynecological oncology center in China. *Ultrasound Obstet Gynecol*. Published Online First: 2019. doi: 10.1002/uog.20363

29 Nohuz E, De Simone L, Chêne G. Reliability of IOTA score and ADNEX model in the screening of ovarian malignancy in postmenopausal women. *J Gynecol Obstet Hum Reprod*. Published Online First: 2019. doi: 10.1016/j.jogoh.2018.04.012

30 Stukan M, Alcazar JL, Gębicki J, *et al.* Ultrasound and Clinical Preoperative Characteristics for Discrimination Between Ovarian Metastatic Colorectal Cancer and Primary Ovarian Cancer: A Case-Control Study. *Diagn Basel Switz*. 2019;9. doi: 10.3390/diagnostics9040210

31 Stukan M, Badocha M, Ratajczak K. Development and validation of a model that includes two ultrasound parameters and the plasma D-dimer level for predicting malignancy in adnexal masses: an observational study. *BMC Cancer*. 2019;19:564.

32 Gaurilcikas A, Gedgaudaite M, Cizauskas A, *et al.* Performance of the IOTA ADNEX Model on Selected Group of Patients with Borderline Ovarian Tumours. *Med Kaunas Lith*. 2020;56. doi: 10.3390/medicina56120690

33 Jeong SY, Park BK, Lee YY, *et al.* Validation of IOTA-ADNEX Model in Discriminating Characteristics of Adnexal Masses: A Comparison with Subjective Assessment. *J Clin Med*. 2020;9. doi: 10.3390/jcm9062010

34 Quaranta M, Nath R, Mehra G, *et al.* Surgery of Benign Ovarian Masses by a Gynecological Cancer Surgeon: A Cohort Study in a Tertiary Cancer Centre. *Cureus*. 2020;12:e9201.

35 Szubert S, Szpurek D, Wójtowicz A, *et al.* Performance of Selected Models for Predicting Malignancy in Ovarian Tumors in Relation to the Degree of Diagnostic Uncertainty by Subjective Assessment With Ultrasound. *J Ultrasound Med Off J Am Inst Ultrasound Med*. 2020;39:939–47.

36 Tug N, Yassa M, Sargin MA, *et al.* Preoperative discriminating performance of the IOTA-ADNEX model and comparison with risk of malignancy index: An external validation in a non-gynecologic oncology tertiary center. *Eur J Gynaecol Oncol*. Published Online First: 2020. doi: 10.31083/j.ejgo.2020.02.4971

37 Butureanu T, Socolov D, Matasariu DR, *et al.* Ovarian masses-applicable iota adnex model versus morphological findings for accurate diagnosis and treatment. *Appl Sci Switz*. Published Online First: 2021. doi: 10.3390/app112210789

38 Czekierdowski A, Stachowicz N, Smoleń A, *et al.* Sonographic Assessment of Complex Ultrasound Morphology Adnexal Tumors in Pregnant Women with the Use of IOTA Simple Rules Risk and ADNEX Scoring Systems. *Diagn Basel Switz*. 2021;11. doi: 10.3390/diagnostics11030414

39 Jiang M-J, Le Q, Yang B-W, *et al.* Ovarian sex cord stromal tumours: analysis of the clinical and sonographic characteristics of different histopathologic subtypes. *J Ovarian Res*. 2021;14:53.

40 Lee SJ, Kim Y-H, Lee M-Y, *et al.* Ultrasonographic evaluation of ovarian mass for predicting malignancy in pregnant women. *Gynecol Oncol*. Published Online First: 2021. doi: 10.1016/j.ygyno.2021.09.007

41 Liu B, Liao J, Gu W, *et al.* ADNEX model-based diagnosis of ovarian cancer using MRI images. *Contrast Media Mol Imaging*. 2021;2021. doi: 10.1155/2021/2146578

42 Nam G, Lee SR, Jeong K, *et al.* Assessment of different NEoplasias in the adneXa model for differentiation of benign and malignant adnexal masses in Korean women. *Obstet Gynecol Sci*. 2021;64:293–9.

43 Peng X-S, Ma Y, Wang L-L, *et al.* Evaluation of the Diagnostic Value of the Ultrasound ADNEX Model for Benign and Malignant Ovarian Tumors. *Int J Gen Med*. 2021;14:5665–73.

44 Poonyakanok V, Tanmahasamut P, Jaishuen A, *et al.* Preoperative Evaluation of the ADNEX Model for the Prediction of the Ovarian Cancer Risk of Adnexal Masses at Siriraj Hospital. *Gynecol Obstet Invest*. Published Online First: 2021. doi: 10.1159/000513517

45 Qian L, Du Q, Jiang M, *et al.* Comparison of the Diagnostic Performances of Ultrasound-Based Models for Predicting Malignancy in Patients With Adnexal Masses. *Front Oncol*. 2021;11:673722.

46 Tavoraitė I, Kronlachner L, Opolskienė G, *et al.* Ultrasound Assessment of Adnexal Pathology: Standardized Methods and Different Levels of Experience. *Med Kaunas Lith*. 2021;57. doi: 10.3390/medicina57070708

47 Behnamfar F, Esmaeilian F, Adibi A, *et al.* Comparison of Ultrasound and Tumor Marker CA125 in Diagnosis of Adnexal Mass Malignancies. *Adv Biomed Res*. 2022;11:18.

48 Budiana ING, Suwiyoga K, Suwardewa TGA, *et al.* Skor assessment of different neoplasias in the adnexa (ADNEX) untuk memprediksi keganasan ovarium di RSUP Sanglah Denpasar. *Intisari Sains Medis*. 2022;13:197–201.

49 Chen G-Y, Hsu T-F, Chan I-S, *et al.* Comparison of the O-RADS and ADNEX models regarding malignancy rate and validity in evaluating adnexal lesions. *Eur Radiol*. 2022;32:7854–64.

50 Esquivel Villabona AL, Rodríguez JN, Ayala N, *et al.* Two-Step Strategy for Optimizing the Preoperative Classification of Adnexal Masses in a University Hospital, Using International Ovarian Tumor Analysis Models: Simple Rules and Assessment of Different NEoplasias in the adneXa Model. *J Ultrasound Med*. Published Online First: 2022. doi: 10.1002/jum.15728

51 Hack K, Gandhi N, Bouchard-Fortier G, *et al.* External Validation of O-RADS US Risk Stratification and Management System. *Radiology*. Published Online First: 2022. doi: 10.1148/radiol.211868

52 He P, Wang J-J, Duan W, *et al.* Estimating the risk of malignancy of adnexal masses: validation of the ADNEX model in the hands of nonexpert ultrasonographers in a gynaecological oncology centre in China. *J Ovarian Res*. 2021;14:169.

53 Hiett AK, Sonek JD, Guy M, *et al.* Performance of IOTA Simple Rules, Simple Rules risk assessment, ADNEX model and O-RADS in differentiating between benign and malignant adnexal lesions in North American women. *Ultrasound Obstet Gynecol*. Published Online First: 2022. doi: 10.1002/uog.24777

54 JIANHONG S, Lei T, Wu L, *et al.* Comparison of performance between O-RADS, IOTA Simple Rules Risk assessment and ADNEX model in the discrimination of ovarian Brenner tumors. Published Online First: 2022. doi: 10.21203/rs.3.rs-2160740/v1

55 Lai H-W, Lyu G-R, Kang Z, *et al.* Comparison of O-RADS, GI-RADS, and ADNEX for Diagnosis of Adnexal Masses: An External Validation Study Conducted by Junior Sonologists. *J Ultrasound Med*. Published Online First: 2022. doi: 10.1002/jum.15834

56 Lam Huong L, Thi Phuong Dung N, Hoang Lam V, *et al.* The Optimal Cut-Off Point of the Andex Model for the Prediction of the Ovarian Cancer Risk. *Asian Pac J Cancer Prev APJCP*. 2022;23:2713–8.

57 Velayo C, Reforma K, Sicam R, *et al.* Prediction of ovarian cancer using a multivariate assay: A randomized controlled trial to improve diagnostic strategies in Filipino women (preliminary results of the overa study). *Int J Gynecol Cancer*. 2020;30:A70–1.

58 Yang Y, Li J, Chen H, *et al.* Assessment of Risk Factors Associated with Severe Endometriosis and Establishment of Preoperative Prediction Model. *Diagn Basel Switz*. 2022;12. doi: 10.3390/diagnostics12102348

59 Czekierdowski A, Stachowicz N, Smolen A, *et al.* Performance of IOTA Simple Rules Risks, ADNEX Model, Subjective Assessment Compared to CA125 and HE4 with ROMA Algorithm in Discriminating between Benign, Borderline and Stage I Malignant Adnexal Lesions. *Diagnostics*. 2023;13. doi: 10.3390/diagnostics13050885

60 Hu Y, Chen B, Dong H, *et al.* Comparison of ultrasound-based ADNEX model with magnetic resonance imaging for discriminating adnexal masses: a multi-center study. *Front Oncol*. 2023;13:1101297.

61 Pelayo M, Pelayo-Delgado I, Sancho-Sauco J, *et al.* Comparison of Ultrasound Scores in Differentiating between Benign and Malignant Adnexal Masses. *Diagn Basel Switz*. 2023;13. doi: 10.3390/diagnostics13071307

62 Rashmi N, Singh S, Begum J, *et al.* Diagnostic Performance of Ultrasound-Based International Ovarian Tumor Analysis Simple Rules and Assessment of Different NEoplasias in the adneXa Model for Predicting Malignancy in Women with Ovarian Tumors: A Prospective Cohort Study. *Womens Health Rep New Rochelle N*. 2023;4:202–10.

63 Wang R, Yang Z. Evaluating the risk of malignancy in adnexal masses: validation of O-RADS and comparison with ADNEX model, SA, and RMI. *Ginekol Pol*. Published Online First: 17 March 2023. doi: 10.5603/GP.a2023.0019

64 Nam G, Lee SR, Jeong K, *et al.* Assessment of different NEoplasias in the adneXa model for differentiation of benign and malignant adnexal masses in Korean women. *Obstet Gynecol Sci*. 2021;64:293–9.

65 Zhang W, Jia S, Xiang Y, *et al.* Factors associated with misdiagnosis of frozen section of mucinous borderline ovarian tumor. *J Int Med Res*. 2019;47:96–104.

66 He P, Wang J-J, Duan W, *et al.* Estimating the risk of malignancy of adnexal masses: validation of the ADNEX model in the hands of nonexpert ultrasonographers in a gynaecological oncology centre in China. *J Ovarian Res*. 2021;14:169.

67 Peng X-S, Ma Y, Wang L-L, *et al.* Evaluation of the Diagnostic Value of the Ultrasound ADNEX Model for Benign and Malignant Ovarian Tumors. *Int J Gen Med*. 2021;14:5665–73.

68 Qian L, Du Q, Jiang M, *et al.* Comparison of the Diagnostic Performances of Ultrasound-Based Models for Predicting Malignancy in Patients With Adnexal Masses. *Front Oncol*. 2021;11:673722.

69 Yang S, Tang J, Rong Y, *et al.* Performance of the IOTA ADNEX model combined with HE4 for identifying early-stage ovarian cancer. *Front Oncol*. 2022;12:949766.

70 Lai H-W, Lyu G-R, Kang Z, *et al.* Comparison of O-RADS, GI-RADS, and ADNEX for Diagnosis of Adnexal Masses: An External Validation Study Conducted by Junior Sonologists. *J Ultrasound Med*. Published Online First: 2022. doi: 10.1002/jum.15834

71 Wang R, Yang Z. Evaluating the risk of malignancy in adnexal masses: validation of O-RADS and comparison with ADNEX model, SA, and RMI. *Ginekol Pol*. Published Online First: 2023. doi: 10.5603/GP.a2023.0019

72 Riley RD, Higgins JPT, Deeks JJ. Interpretation of random effects meta-analyses. *BMJ*. 2011;342:d549.

73 McGuinness LA, Higgins JPT. Risk-of-bias VISualization (robvis): An R package and Shiny web app for visualizing risk-of-bias assessments. *Res Synth Methods*. 2021;12:55–61.

74 Wolff RF, Moons KGM, Riley RD, *et al.* PROBAST: A Tool to Assess the Risk of Bias and Applicability of Prediction Model Studies. *Ann Intern Med*. 2019;170:51.

75 Moons KGM, Wolff RF, Riley RD, *et al.* PROBAST: A Tool to Assess Risk of Bias and Applicability of Prediction Model Studies: Explanation and Elaboration. *Ann Intern Med*. 2019;170:W1.
